# Robust Prediction of Expression Differences among Human Individuals Using Only Genotype Information

**DOI:** 10.1371/journal.pgen.1003396

**Published:** 2013-03-28

**Authors:** Ohad Manor, Eran Segal

**Affiliations:** 1 Department of Computer Science and Applied Mathematics, Weizmann Institute of Science, Rehovot, Israel; 2 Department of Molecular Cell Biology, Weizmann Institute of Science, Rehovot, Israel; Georgia Institute of Technology, United States of America

## Abstract

Many genetic variants that are significantly correlated to gene expression changes across human individuals have been identified, but the ability of these variants to predict expression of unseen individuals has rarely been evaluated. Here, we devise an algorithm that, given training expression and genotype data for a set of individuals, predicts the expression of genes of unseen test individuals given only their genotype in the local genomic vicinity of the predicted gene. Notably, the resulting predictions are remarkably robust in that they agree well between the training and test sets, even when the training and test sets consist of individuals from distinct populations. Thus, although the overall number of genes that can be predicted is relatively small, as expected from our choice to ignore effects such as environmental factors and *trans* sequence variation, the robust nature of the predictions means that the identity and quantitative degree to which genes can be predicted is known in advance. We also present an extension that incorporates heterogeneous types of genomic annotations to differentially weigh the importance of the various genetic variants, and we show that assigning higher weights to variants with particular annotations such as proximity to genes and high regional G/C content can further improve the predictions. Finally, genes that are successfully predicted have, on average, higher expression and more variability across individuals, providing insight into the characteristics of the types of genes that can be predicted from their *cis* genetic variation.

## Author Summary

Variation in gene expression across different individuals has been found to play a role in susceptibility to different diseases. In addition, many genetic variants that are linked to changes in expression have been found to date. However, their joint ability to accurately predict these changes is not well understood and has rarely been evaluated. Here, we devise a method that uses multiple genetic variants to explain the variation in expression of genes across individuals. One important aspect of our method is its robustness, in that our predictions agree well between training and test sets. Thus, although the number of genes that could be explained is relatively small, the identity and quantitative degree to which genes can be predicted is known in advance. We also present an extension to our method that integrates different genomic annotations such as location of the genetic variant or its context to differentially weigh the genetic variants in our model and improve predictions. Finally, genes that are successfully predicted have, on average, higher expression and more variability across individuals, providing insight into the characteristics of the types of genes that can be predicted by our method.

## Introduction

Variation in gene expression among human individuals plays a key role in the susceptibility to different diseases and phenotypes [1]–[7]. Some of this variation has been linked to genetic variation that exists among individuals [8]–[16]. For this reason, a major goal is to predict gene expression changes among individuals based on their genetic sequence variation. Ideally, if we could predict the expression profile of genes for each individual, we could estimate this individual's risk for developing certain diseases; but unfortunately, our current ability to make such predictions is poor.

A popular approach to study the relationship between genetic variation and expression variation is by using a mapping approach, where the expression of every gene is treated as a quantitative trait and one searches for genetic changes that are significantly correlated with the expression changes of each gene. A dominant method is to fit a linear regression model from the minor allele count of single nucleotide polymorphisms (SNPs) to the expression level of a gene across different individuals. Under some assumptions, a p-value for the resulting correlations can be computed and after correcting for multiple hypotheses testing, the set of SNPs that are significantly associated with the expression changes of every gene can be identified. Using this approach researchers were able to examine all of the SNPs measured in the human genome for significant associations in an unbiased way, resulting in many potential effectors of gene expression [9], [10], [17], [18].

A major issue in the above analyses is low statistical power for detecting associations that arises from the large number of SNPs examined. This means that SNPs with a low correlation to expression will not be detected as possible effectors. However, since the degree of correlation of SNPs to expression is associated with their genomic features such as location (e.g., distance from the transcription start site) or function [9]–[16], [18]–[20] (e.g., within transcription factor binding sites), several studies used genomic features of SNPs as a prior on the probability of their functional importance within a Bayesian framework [13], [19], [20], resulting in additional statistical power to detect associated SNPs in particular genomic regions.

A second issue is that the above methods examine each SNP in isolation. Thus, they cannot detect combinatorial associations between multiple SNPs and expression changes. Several methods tried to address this issue by examining multiple SNPs at the same time using ridge-regression [21], a Bayesian approach [22], or multiple interval mapping [23], and identified statistically significant epistatic interactions that are not found using the single SNP approaches.

Collectively, the above methods focused on mapping SNPs that are significantly correlated with expression and have identified many interesting associations between expression changes across genes and genotype data. However, since their goal was to identify statistically significant associations of single SNPs, they do not consider multiple-SNP models, where combinations of SNPs and SNPs with small effects could play a role in generating the predictive model. One work attempted to devise a multiple-SNP predictive model using a cross validation scheme, whereby a model whose parameters were fitted from the data of a subset of the individuals is tested for its ability to predict the expression of the remaining unseen individuals [24]. This work also integrated genomic features of SNPs, using them as priors in a regularized linear model, and found SNPs located in certain regions of the gene to be more predictive of gene expression variation. However, in addition to the SNP information, this work also used the expression value of other genes for the prediction task, making it difficult to assess which part of the reported predictive power comes from SNP information and which from the expression of correlated genes.

Here, we set out to develop an algorithm that predicts changes in gene expression among human individuals using only their genetic variation. Notably, this goal is different from that of the above studies that focus on mapping associated single SNPs. Accordingly, rather than providing p-values for SNP associations, we evaluate the quality of our algorithm by its ability to predict the gene expression levels of unseen human individuals given only their genotype information. Contrary to the single SNP association studies mentioned above, we use multiple SNPs to predict the expression of every gene, but to avoid overfitting only SNPs that are in the genomic vicinity of the predicted gene are used. We also incorporate a comprehensive set of genomic and functional features for each SNP, by allowing the algorithm to use this meta-information to weigh the importance of each SNP in the prediction.

We evaluated the ability of several algorithms to predict gene expression data from immune precursor cells of four different human populations for which corresponding SNP data is available [8], [10]. Clearly, this prediction task is very difficult for several reasons, including noise in the data and missing SNPs, and because the expression of many genes is determined by environmental factors and by *trans*-acting SNPs that we do not consider. Nevertheless, comparing two different multi-SNP algorithms and one single-SNP algorithm, we find that both a variation of the K-nearest neighbor (KNN) algorithm [25], [26] and a regularized linear model achieve high correspondence between their predictions on the training data and those on the held out test data. We also show that combining their predictions into a single model improves the predictions on held-out test data, as the two models explain different aspects of the expression data. As expected, the total number of genes whose expression can be accurately predicted using their proximal SNP data is relatively small, but the high robustness of our algorithm means that it can determine the quality of the predictions in advance with high accuracy.

As we had four different populations in the data, we evaluated the predictions on several different cross validation schemes. Notably, we found that incorporating individuals from other populations improves the predictions of a given population compared to only using the individuals of the predicted population, suggesting that some of the SNPs that are associated with expression changes are shared across populations. We also found that our algorithm achieves similar performance when it attempts to predict the expression of genes from one population using only the data of other populations.

Taken together, our work provides concrete means by which some of the expression variation among human individuals can be predicted with a low false positive rate using only their genotype data, suggesting that the time may be ripe for a broader examination of this important prediction task, perhaps while incorporating additional features such as *trans*-acting SNPs.

## Results

### Experimental Data and Cross Validation Schemes

As the data for our study, we used the HapMap Phase II dataset [8], consisting of 210 unrelated individuals from four distinct populations of European (CEU), African (YRI), Chinese (CHB), and Japanese (JPT) origins, for which corresponding expression measurements from immune precursor cells are available for 15,439 genes [10]. We defined a prediction task for each such gene using only its set of proximal *cis-*SNPs, defined as those SNPs that reside inside the body of the gene or in the 100 kb upstream and downstream regions flanking the gene. This resulted in a total of ∼4.7 M *cis*-SNPs across all 15,439 genes, for an average of ∼304 SNPs per gene.

**Figure 1:**
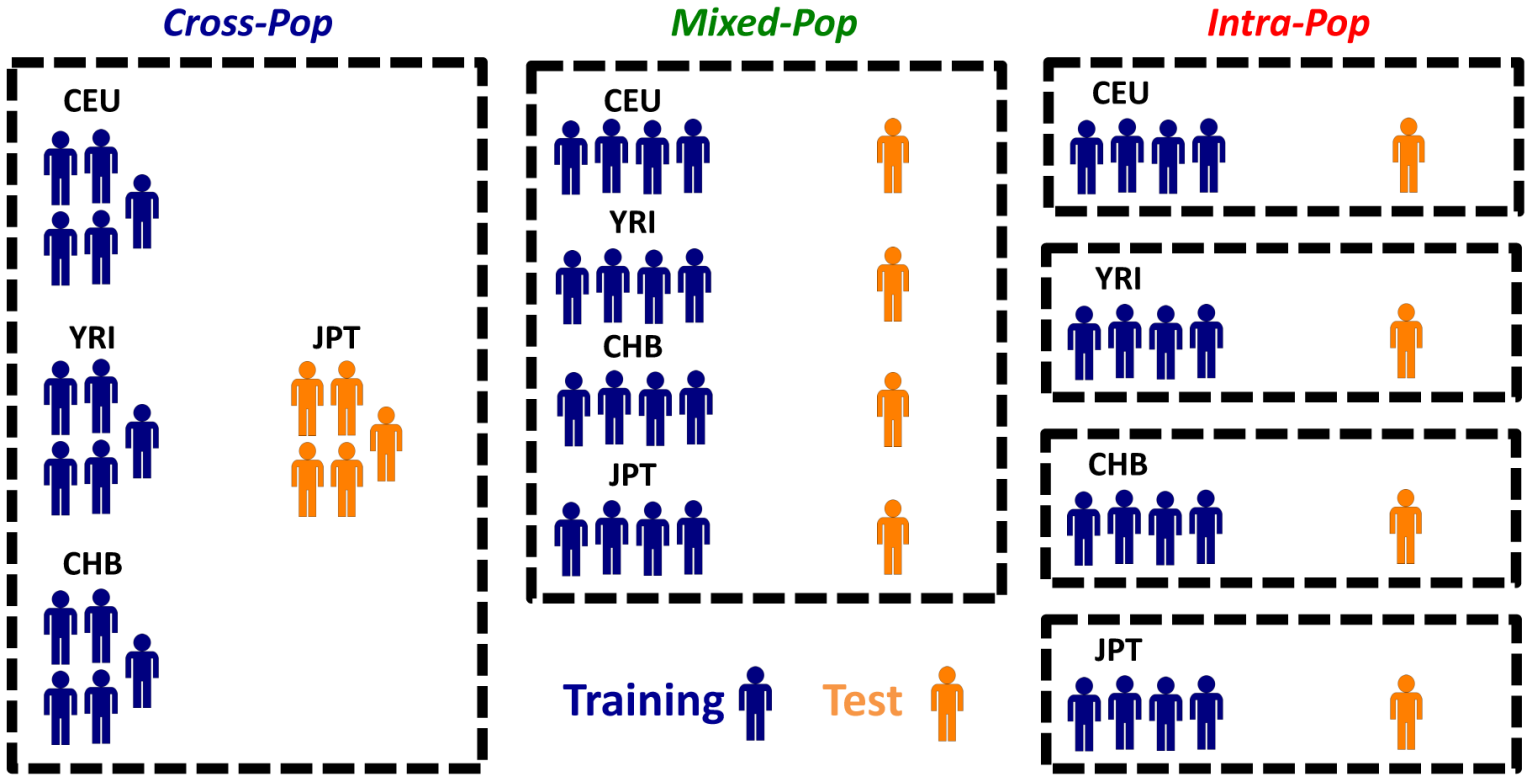
Different cross-validation schemes used throughout the paper. Shown are the three different cross validation schemes that we used throughout the paper. In the *Cross-Pop* scheme (left column), a 4-fold cross validation scheme is used, whereby in each partition three of the four populations are used as the training set (blue), and one population is held out as a test set (colored orange). In the *Mixed-Pop* scheme (middle column), individuals from all populations are randomly partitioned into five equally sized sets, and each of the five possible choices of four sets is then used as training, with the fifth held out set used as the test set. Finally, in the *Intra-Pop* scheme (right column), a standard five fold cross validation scheme is applied separately to each population (i.e., the training and test set always consist of individuals from the same population).

To assess whether the predictive power comes from using individuals of the same population or those of other populations, we used three different cross-validation (CV) schemes throughout the paper (Figure 1). In the first *Cross-Pop* scheme, three populations serve as training data, and the remaining population is used as the test data. In the second *Mixed-Pop* scheme, the populations are mixed such that the training and test data both contain individuals from all four populations. Finally, in the third *Intra-Pop* scheme, the partition to training and test is done separately for each population, such that there is a separate test data for each of the four populations that is predicted using a model learned only from the remaining individuals of the tested population. The prediction task of the *Cross-Pop* scheme may be expected to be the hardest, since the parameters are learned using only the data of other distinct populations, which requires the SNPs affecting expression to be shared across populations. Conversely, in the *Intra-Pop* scheme, the model can select different SNPs for each population, if each population has a different SNP affecting expression. This may improve the predictions but can also lead to overfitting of the training data. Finally, in the *Mixed-Pop* scheme, although SNPs have to be shared across populations, having representatives from all populations in the training and test sets may improve the robustness of the model.

### Robust Prediction of Expression of Unseen Individuals from Multiple SNPs

Most studies of the relationship between genetic and expression variation have focused on identifying single SNPs that are significantly correlated with gene expression changes [8]–[16]. Thus, the question of whether multiple SNPs can be used jointly to improve predictions of gene expression remains largely unexplored, and hence, we sought to devise an algorithm that can robustly predict the expression of unseen test individuals using multiple SNPs. Given that we restricted ourselves to *cis-*SNPs and that many expression changes are determined by environmental factors or by *trans*-SNPs, this prediction task is difficult and even the optimal predictor would not be able to explain most of the observed variation in expression. Rather, we strive for a robust predictor that will have good agreement between the training and test data, such that it can determine its performance on held out individuals in advance using only the individuals given to it during the training phase.

A predictive model based on a single SNP (*single-SNP model*) will have only two parameters for regression, namely the slope and the intercept. Therefore, we decided to compare this model with two different multi-SNP models that vary in the number of parameters they use. The first model is based on the K-nearest neighbor (KNN) algorithm, which predicts the expression level of an unseen individual based on the measured expression values of the K individuals in the training set whose genotypes are most similar to it [25], [26]. In the KNN algorithm, similarity is measured by a distance metric, which we defined in our case as the absolute difference in minor allele counts summed over all *cis-*SNPs of the tested gene. We chose the KNN algorithm due to its inherent property of generating its predictions using the measured values of those individuals that are closest to it in feature (genotype) space, rather than by attempting to construct a generative model of its predicted values. This property allows the KNN model to use multiple SNPs in the prediction, while using only a single parameter, K (the number of neighbors) and it is thus similar to the single-SNP model in terms of the parameter space it uses. The second multi-SNP model we consider is a regularized (elastic-net) linear regression model [27] that has one weight parameter for each SNP, thereby increasing the number of parameters to the number of SNPs used by the model. In order to increase robustness, we employed an internal training cross-validation procedure to determine the strength of the regularization and the training performance (see Methods). We also combined the two multi-SNP models into a single model by averaging their predictions for each individual, since we hypothesized that the different assumptions and number of parameters employed by the two models will capture different aspects of the data and hence their combination could improve the predictions.

We tested these different models in each of the three different cross validation schemes. For all models, in each cross validation partition we used the training data to learn the parameters for each gene, which maximize the reduction in variance achieved by the corresponding predictor (i.e., regressor or KNN, see Methods). We measure this reduction using R^2^, defined as the proportion of variance in the data (either training or test) explained by the predictor.

**Figure 2:**
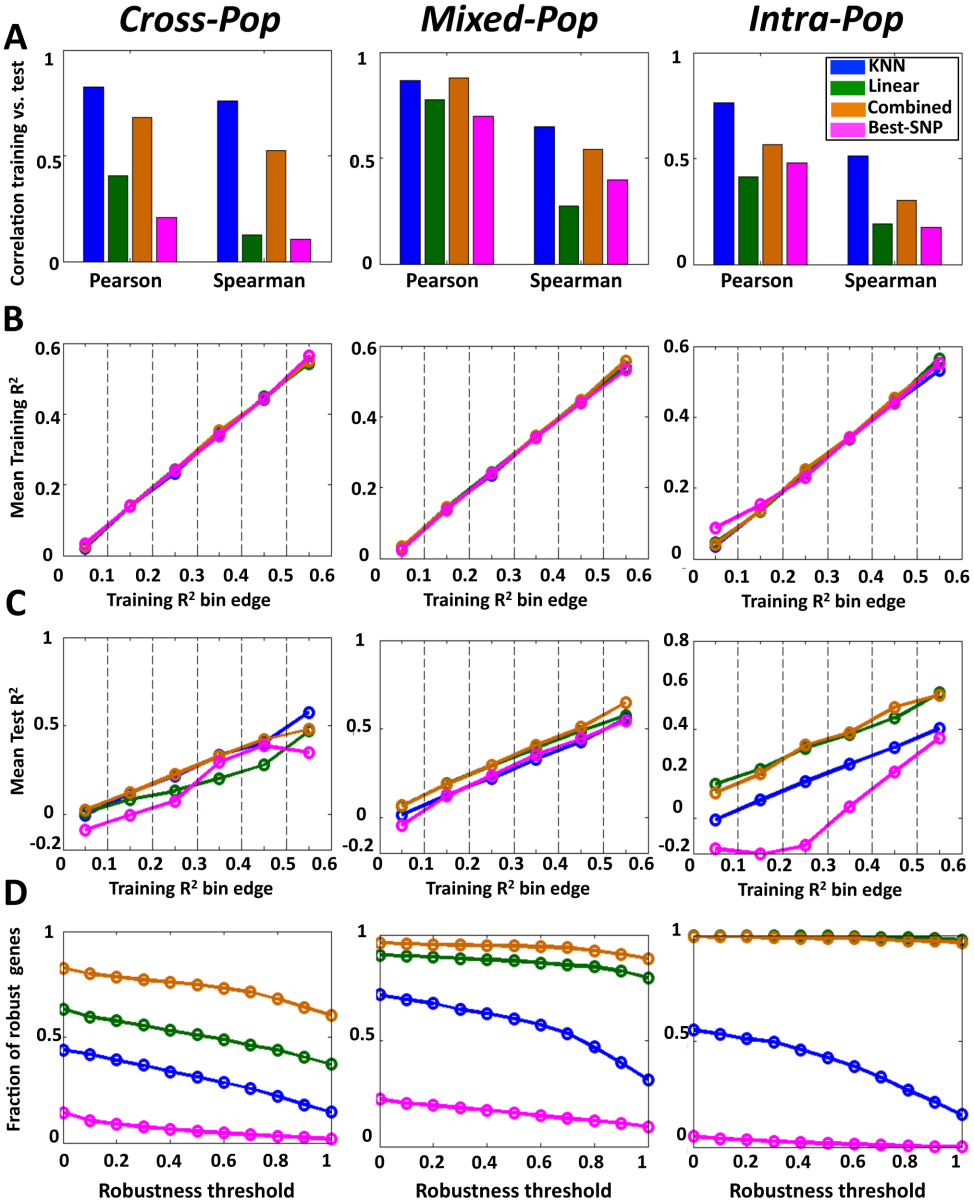
Multi–SNP algorithms predict expression of unseen test individuals with high robustness. (A) Predictions of the KNN algorithm are more correlated between the training and test sets compared to the linear and best-SNP models. For the KNN-based algorithm, the regularized linear regression model, and the single best-SNP model that uses the most correlated SNP, shown are the Pearson and Spearman correlation coefficients between the training and test R^2^ across all genes and all three cross validation schemes. (B) For each of the three models and every cross-validation scheme, genes were binned by training R^2^. Shown is the mean training R^2^ of the genes in every bin. (C) For genes with the same training R^2^ in each model, the multi-SNP algorithms show higher test R^2^. For the same bins from (B), shown is the mean test R^2^ of the genes in every bin. (D) The combined algorithm is more robust than the all other models. For each model and every cross validation scheme, genes with training R^2^>0.05 were extracted. For each such set of genes, shown is the fraction (y-axis) of genes whose test R^2^ by each respective model and cross validation scheme were within some fraction (x-axis, robustness threshold) of their training R^2^.

Notably, for all multi-SNP models, we found high correlations between their predictions on the training data and their predictions on the test data in all three cross validation schemes (e.g., Pearson correlations of 0.57–0.88 between training and test results across mean cross-validation values of 15,439 genes for the combined model). In nearly all cases, these correlations between the training and test predictions were higher than those achieved by the single-SNP model. This improved performance of the multi-SNP models was most notable in the hardest *Cross-Pop* prediction scheme, where the KNN model had 0.83 Pearson correlation, compared to the 0.21 of the single-SNP model (Figure 2A, Figure S1, Figure S2). The improved performance of the multi-SNP models was also evident when binning the genes according to their training performance in each algorithm and then comparing the R^2^ that each algorithm achieves on the test data (Figure 2B, 2C). For example, for genes with training R^2^ between 0.2 and 0.3 (i.e., 20–30% of the expression variation of the training set is explained), the KNN, linear and combined models achieve an average test R^2^ of 0.21, 0.13 and 0.22, respectively, whereas the single-SNP model achieves test R^2^ of 0.075 (Figure 2C, *Cross-Pop*).

The R^2^ values above represent an average over multiple genes, and thus, they may not be indicative of the R^2^ of individual genes. As one way to examine performance at the single gene level, we defined a robustly predicted gene as one with test R^2^ equal to or larger than some threshold fraction of its training R^2^ (only genes with training R^2^≥0 are considered). Here too, we found the multi-SNP models to have a much higher fraction of robustly predicted genes across all thresholds and cross-validation schemes as compared to the single-SNP model (Figure 2D). Notably, the differences in this analysis are even more striking than those suggested by the average R^2^ values, where for example, at a robustness threshold of 0.5 in the *Cross-Pop* scheme (i.e., test R^2^ of a gene is at least half of its training R^2^), 31%, 51% and 75% of the genes are robustly predicted by the KNN, linear and combined models, respectively, compared to only 5% for the single-SNP model.

As expected from this hard prediction task, the overall number of genes that can be well predicted is relatively small (Table 1, Tables S1 and S2, and Figure S3). For example, 364/15,439 (2.4%) and 529/15,439 (3.4%) of the genes have training and test R^2^ above 0.1 in the Cross-pop and Mixed-pop CV schemes, respectively. However, the robust nature of our algorithms means that the number of false positive predictions will be relatively small, i.e., the algorithm knows in advance which genes will be well predicted. This high degree of robustness persists and is most impressive in the *Cross-Pop* scheme, where the expression levels of an entire population is predicted without using any individual from that population.

**Table 1:** Number of genes that pass R^2^ threshold.

R^2^ threshold	KNN	Elastic-Net	Combined	Single SNP	All models
0.05	273/622	388/640	401/493	533/2659	700/2730
0.1	152/350	239/389	213/265	272/685	364/753
0.2	47/125	114/189	82/105	100/179	143/245
0.3	18/57	54/94	35/42	50/72	73/115
0.4	8/29	32/55	15/20	14/25	36/62
0.5	4/17	14/28	6/11	5/7	16/29

Models are: K-nearest-neighbor (KNN); Regularized Linear model (Elastic-Net); single best-SNP (Single SNP) or in any one of them (All models). Shown are the number of genes that pass training and test R^2^ threshold (test/training) using the different models in the Cross-Pop cross-validation scheme.

When comparing the overlap between top-predicted genes (in test data) for the different models, we find that ∼50% of the top-predicted genes overlap across all models (Table 2, Tables S3 and S4, top-predicted gene lists appear in Tables S5, S6, S7, S8, S9, S10, S11, S12, S13). This result suggests that some genes can be well predicted regardless of the selected model (i.e., even a single-SNP does relatively well), but for other genes the selection of the model type, i.e., linear (Elastic-Net) or non-linear (KNN) can have a large effect on our ability to correctly predict held-out test data.

**Table 2:** Number of top predicted genes that overlap between different models.

Top genes	KNN-EN	KNN-SS	EN-SS	All models
10	3	3	6	3
20	10	8	14	7
50	29	26	39	24
100	52	47	79	43
200	119	108	152	97

Models are: K-nearest-neighbor (KNN); Regularized Linear model (EN); single best-SNP (SS) or in any one of them (All models). Shown are the numbers of top predicted genes that overlap between the different models in the Cross-Pop cross-validation scheme.

Although we found the multi-SNP models to have better robustness in terms of agreement between training and test results, when comparing the best model across all predicted genes, we find that on average, ∼33% of the genes are best predicted by the single-SNP model, while the other ∼66% are best predicted by one of the multi-SNP models (Table S14). This result emphasizes the fact that in different scenarios, different models perform best, and the best approach would probably make combined use of all models.

In conclusion, these results demonstrate that multi-SNP models, including both the KNN-model that uses a single parameter and the regularized linear model that uses multiple parameters, are overall more robust than the single-SNP model, and can outperform it for most genes. In addition, we found that a model that combines the two multi-SNP models can be beneficial in terms of prediction robustness as compared to the individual models.

### Integration of Genomic Features of SNPs Improves the Predictions on Unseen Data

Despite the robust predictions of our KNN-based algorithm, in the distance metric that it employs all SNPs contribute equally. Since SNPs can vary greatly in their correlation to the expression of the nearby gene, and the degree to which their expression correlations on the training set match their correlations on the test set, we asked whether SNPs with better agreement have particular properties. We reasoned that if that were the case, then we might be able to improve performance by using this information to assign differential weights to SNPs within the distance metric. Motivated by studies demonstrating that SNPs that are closer to the transcription start site or SNPs that are located within 3′ UTRs have higher expression correlations [10], [24], [27], we generated a comprehensive set of 117 genomic features for every SNP (Table S15). In addition to previously examined annotations such as the location of the SNP (e.g., within a UTR, intron, or gene body), and its distance from the transcription start and end sites, we also added features such as the conservation and G/C nucleotide content around the SNP, and whether or not the SNP resides within predicted microRNA or transcription factor binding sites. We also added features based on chromatin immunoprecipitation experiments, such as whether or not a SNP is located in a genomic region marked with a specific histone methylation or acetylation.

**Figure 3:**
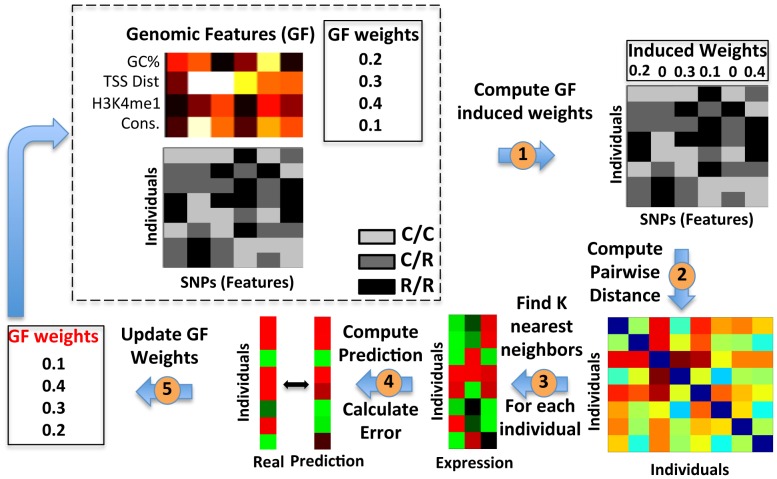
Extending the K-Nearest-Neighbor algorithm to integrate genomic features. Illustration of our extension to the KNN algorithm that integrates genomic features. The algorithm starts with two constant datasets, and a genomic feature weight vector (dashed box) that it updates. The constant datasets consist of the SNP matrix (bottom matrix), in which every entry is colored by the allele composition of the individual (‘c’, common; ‘r’, rare), and the genomic feature matrix (top matrix), in which every entry represents the value of the genomic feature for the corresponding SNP. The genomic feature weight vector represents the relative weight or importance of each feature. The algorithm updates this weight vector iteratively. First, we compute the SNP induced weights vector, by multiplying the genomic feature weight vector with the genomic feature matrix (1). Next, we compute the pairwise distances between every two individuals (rows), by summing the differences in allele composition across all SNPs, where each SNP is weighed by its corresponding induced weight (2), resulting in a pairwise distance matrix (bottom right). This pairwise distance matrix is then used to find the *k* nearest neighbors for each individual, i.e., those *k* individuals with the smallest distance from each individual (3). Then, we average their expression values to generate a prediction for this individual. We apply this process to each individual, and obtain an expression prediction vector for all individuals (4). Finally, we compare the prediction to the true expression vector, and use this difference to update the genomic feature weight vector for the next iteration, by selecting the weight vector that minimizes this difference the most from among a candidate pool. (5). We iterate until convergence to a local maximum, whereby changing the genomic feature weight vector does not further improve the prediction on training data.

Next, we extended the KNN algorithm to integrate genomic features into the model by assigning a weight to each genomic feature representing its relative importance. We then updated the distance metric employed by the KNN algorithm such that for each SNP that it sums over when computing the distance between the genotypes of two individuals, it weighs the absolute difference of the SNP's allele counts between the two individuals by the weighted sum of the SNP's genomic features (Figure 3). The extended algorithm learns the weights of the genomic features in an iterative manner, by updating the weight of each genomic feature in a way that maximizes the training R^2^ and thus improves the overall expression predictions of the algorithm (Methods). The greedy nature of the algorithm ensures that it improves in each iteration until a local maxima is reached in which changes to the weights of the genomic features do not further improve the training R^2^.

**Figure 4:**
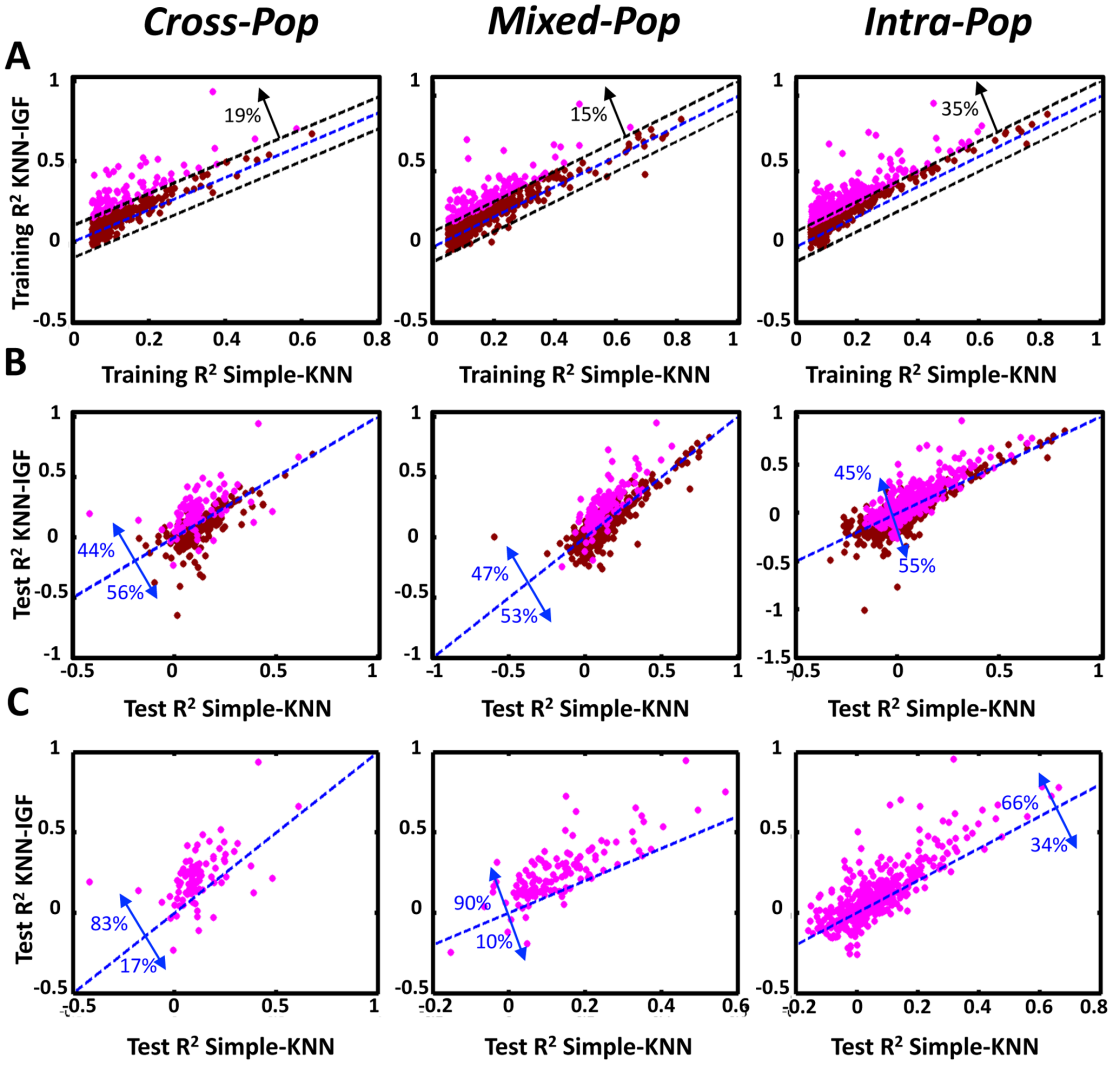
Integrating genomic features improves the performance of the KNN algorithm. (A) Shown is a comparison of the training R^2^ values for genes in either the basic KNN algorithm (x-axis) or the extension of the KNN algorithm that integrates genomic features (*KNN-IGF*, y-axis). Only genes that have training R^2^≥0.05 in the basic KNN were included in the analysis. Results are shown for each cross validation scheme, along with the fraction of genes with improved training R^2^ of 0.1 or more in KNN-IGF (pink). (B) Shown is a comparison of the test R^2^ values for same genes in either the basic KNN algorithm (x-axis) or the KNN-IGF (y-axis). Results are shown for each cross validation scheme, along with the fraction of genes that better predicted in the basic KNN (under the blue line) or in KNN-IGF (above blue line). (C) Same as (b), but filtered for genes whose training R^2^ when integrating genomic features was at least 0.1 higher than the training R^2^ of the basic KNN algorithm. As seen, most genes with improved training R^2^ also have better test R^2^.

**Figure 5:**
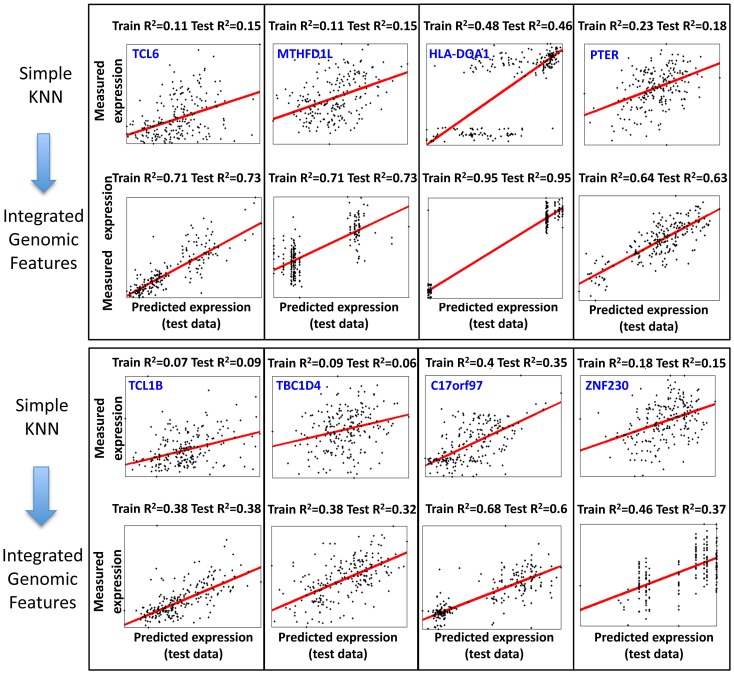
Examples of predictions for genes with large training R^2^ improvement when integrating genomic features. Shown are the 8 genes for which the KNN algorithm that integrated genomic features resulted in the greatest R^2^ improvement in the training data as compared to the basic KNN algorithm. Each plot shows the expression predictions on the test data (x-axis) versus the measured expression values (y-axis), where every dot represents one of the 210 individuals. The regression curve for each plot is also shown (red curve) along with the gene name, and training and test R^2^ values.

To test whether this extended algorithm can improve the predictions obtained by the simple-KNN algorithm, we applied it to all genes whose training R^2^ in the simple-KNN algorithm was above 0.05 in each of the cross-validation schemes. For most genes (65–85%, depending on the cross validation scheme, Figure 4A), integrating genomic features did not result in large improvements of the training R^2^ obtained by the simple-KNN algorithm. Correspondingly, the test R^2^ values improved for only ∼45% of these genes (Figure 4B). However, for most (66–90%) of the genes for which integrating genomic features resulted in a substantial (above 0.1) increase in their training R^2^, the test R^2^ also increased, and the average test R^2^ increase of these genes was ∼0.1 in all three cross-validation schemes (Figure 4C, Figure 5). Thus, although integrating genomic features improves the training predictions for only a subset of genes (15–35%, depending on the cross-validation scheme), the extended algorithm maintains the robustness property of the simple KNN algorithm, whereby it can identify in advance which genes will be better predicted on unseen individuals.

### Genomic Features Related to Regulation and Activation Are Significantly Enriched in Both Multi–SNP Models

**Figure 6:**
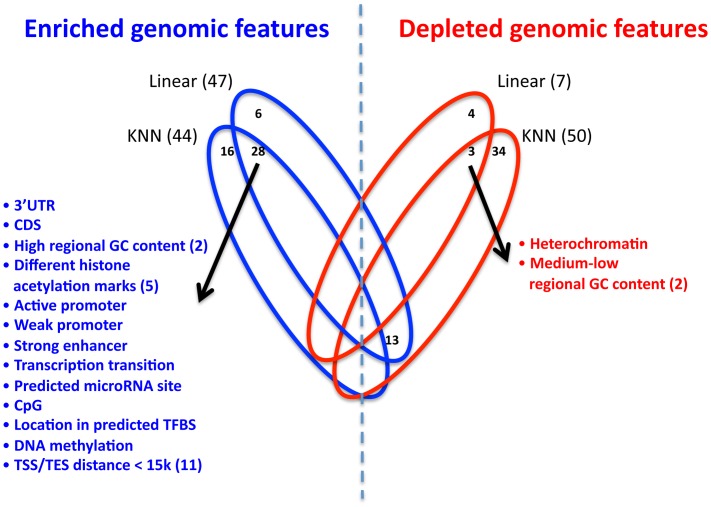
Enrichment and depletion of different genomic features in the multi–SNP models. Venn diagram showing the number of significantly enriched (blue) or depleted (red) genomic features in the extended KNN algorithm and the regularized linear model. For each model, the number of significant genomic features appears in parenthesis, and non-zero intersections are shown in the appropriate location. The list of respective genomic features (enriched or depleted) is depicted, and the number in parenthesis represents the actual number of different genomic features in the group.

Having integrated the SNP genomic features into our extended KNN algorithm, we next examined which genomic features are significantly enriched or depleted in it. The extended KNN model explicitly assigns different weights to the different genomic features, where the weights are learned for each gene separately. Therefore, for each genomic feature, we can compute the significance of its enrichment or depletion in having a non-zero weight across all genes. We find that 44/117 (38%) and 50/117 (43%) of the genomic features are significantly enriched or depleted in the extended KNN model, respectively (Figure 6, P<0.01 in all CV partitions, hypergeometric test, FDR [28] correction). In contrast, the regularized linear model does not use the genomic features information in the learning process. However, as different SNPs were assigned different weights, we can determine whether SNPs that share a certain genomic feature are given a non-zero weight significantly more than expected by chance, or significantly less. We find that 47/117 (40%) and 7/117 (6%) of the genomic features are significantly enriched or depleted in the regularized linear model, respectively.

Since the two multi-SNP models were independently learned, we also examined their intersections of enriched or depleted genomic features. We found that 28 genomic features are enriched in both models, which is a significantly larger intersection than expected by chance (P<10^−4^, hypergeometric test). Among these enriched genomic features are all distance bins of 15 kb or smaller from TSS or TES, suggesting that sequence variation in these regions is most important for regulation by *cis*-SNPs, consistent with the higher known expression correlation of proximal SNPs [10], [13] (Figure 6, full list of enriched/depleted genomic features in Table S16). In addition, the genomic feature of high (>0.7) regional GC content (50–100 bp region flanking SNP) was also enriched in both models. High GC content was shown to correlate with higher nucleosome occupancy and regulatory function in humans [29], which may explain why SNPs in these regions are more predictive of expression changes. SNPs in predicted binding sites for both transcription factors and microRNAs were also enriched in both models, as may be expected, since such SNPs may affect the likelihood of binding of the transcription factor [30] or microRNA [31].

Among the various post-translational histone modifications included in our genomic feature set (20 methylations and 18 acetylation marks, Table S15), only 5 histone acetylation marks were significantly enriched in both models (Table S16). Notably, all 5/5 (100%) of these acetylation marks are enriched in enhancers, compared to 11/18 (61%) of all histone acetylation marks [32], suggesting that SNPs in enhancer elements may have a larger effect on gene expression. Next, we examined genomic features that are significantly depleted in both the extended KNN and the linear models. We find only 3 such genomic features, consisting of medium-low regional GC content, and heterochromatin marks. These results suggest that areas with lower regulatory capacity have a smaller effect on variation in gene expression.

To conclude, the analysis of the genomic features that both multi-SNP models selected in an unbiased manner as being more (or less) important for the predictions suggests that genetic variation in active and regulatory regions are more likely to affect expression compared to variation in other regions.

### Genes That Are Well Predicted Have Higher Expression Levels and More Variability

To gain further insight into properties of genes that may allow for good predictions, we searched for commonalities in genes that our algorithms predicted successfully. We arbitrarily defined well-predicted genes as those with both training and test R^2^ above 0.05 in any of the three-cross validation schemes, resulting in 850 such genes. Notably, we found that the KNN algorithm selected significantly fewer neighbors when predicting these genes (K = 11.9 vs. 15.2 for the predictable vs. non-predictable genes, respectively, P<10^−200^, Table 3 and Figure S4), and that predictable genes had higher absolute expression levels (8.2 vs. 7.7, P<10^−18^, Table 3) and were more variable, as measured by their coefficient of variation (standard deviation divided by mean expression, 0.039 vs. 0.027, P<10^−40^, Table 3). Genes with lower expression and less variability are noisier and their measured variation may thus not represent true variation, which may explain why our algorithm is less successful at predicting them. We also found that predictable genes have on average fewer *cis-*SNPs (291 vs. 307, P<0.002), but their average information content per SNP, measured by the SNP's entropy, is higher (0.44 vs. 0.42, P<10^−7^).

**Table 3:** Comparison between predictable and unpredictable genes.

Feature	Predictable	Unpredictable	P-value
Number of Neighbors (*K*)	11.9	15.2	1.00E-200
Abs. Expression	8.2	7.7	5.80E-19
Coefficient of Variation	0.039	0.027	4.20E-41
Num. SNPs	291	307	0.002
SNP entropy	0.44	0.42	8.00E-08

Feature	Predictable (KNN-IGF)	Predictable (Simple-KNN)	P-value
Number of Neighbors (*K*)	10.1	10.5	NS
Abs. Expression	8.2	8.3	NS
Coefficient of Variation	0.04	0.04	NS
Num. SNPs	307	255	0.0004
SNP entropy	0.44	0.44	NS

Compared features are the number of neighbors selected by the model (i.e., k), the absolute expression level of the gene, the coefficient of variation of the gene (i.e., standard variation normalized by mean), the number of SNPs per gene and the sum of entropy of SNPs per gene. Shown are the average values for each set of genes (850 predictable genes vs. 14,587 unpredictable genes in the top rows, and 412 K-nearest-neighbor-integrating-genomic-features (KNN-IGF) predictable genes vs. 90 simple-KNN predictable genes in the bottom rows), and the p-value calculated by Student's t-test (NS is non-significant).

Finally, comparing genes that are predicted better by the extended KNN algorithm integrating genomic features (*KNN-IGF*, 412 genes) to those that are predicted better by the simple-KNN algorithm (90 genes), we found that genes that are better predicted in the extended version have more SNPs (307 vs. 255, P<0.0004) and similar information content per SNP (0.44 in both cases). This suggests that by integrating genomic features and weighting the relevance by various genomic annotations, we can integrate more features (SNPs) without overfitting.

We next asked whether predictable genes are enriched for particular biological functions or processes by testing whether they are enriched with particular GO annotations. After correcting for multiple hypotheses testing using FDR [28], we found 18 GO annotations that were significantly enriched in the 850 genes that were better predicted (see Table S17 for the full list). Of these, the most notable enrichments were immune response categories, such as MHC protein complex and antigen processing and presentation (P<10^−4^ and P<10^−3^, respectively), which may be expected given that the expression measurements were done in a lymphoblastoid cell line. This result is consistent with the finding that SNPs can effect expression in a cell-type specific manner [33], and with another study that found enrichment for immune-related phenotypes in the genes associated with SNPs that have high expression correlation in these cell lines [7]. Since our algorithm only used *cis-*SNPs for its predictions, these results suggest that *cis-*variation may underlie part of the expression variation of immune response genes.

High expression of a gene in a particular cell type suggests regulation in that cell type. Thus, our ability to better predict highly expressed genes suggests that predicting the expression variation of a gene based on its *cis-*SNPs requires that gene to be regulated in the measured cell type. Clearly, even if the variation that exists in the *cis-*regulatory sequence of a particular gene affects its expression, the effect will not be observed in cell types in which that gene is not regulated.

## Discussion

Here, we devised two multi-SNP algorithms for predicting gene expression variation among human individuals using only genotype information. The algorithms use information from multiple SNPs in the local vicinity of the predicted gene and we also present a combination of both algorithms and an extension that incorporates heterogeneous sources of genomic annotations of SNPs and assigns higher relevance to variation in SNPs with particular annotations. Notably, we show that our algorithms can predict the expression of genes of unseen individuals with remarkable robustness even when the training set consists only of data from populations different than that of the predicted individuals. In fact, a subset of genes are predicted better by our algorithm when incorporating information from populations other than that of the predicted individuals, suggesting that part of the predictive sequence variation is shared across different populations and may be hard to distill using only individuals from the same population.

The overall number of genes that can be accurately predicted by our algorithms is relatively small, which is expected given that we only consider *cis*-SNPs and ignore trans sequence variation and environmental factors. However, the robust nature of our algorithms means that although relatively small in number, the genes that can be successfully predicted can be known in advance, as well as the quantitative degree to which they will be predicted. We show that both a regularized linear regression model and a KNN-based model that use multiple SNPs are more robust than a single-SNP based model, especially when predicting expression across populations. We also show that combining these two models into a single model results in improved predictions on held-out test data. In addition, although multi-SNPs models are more robust, we found different genes to be best predicted by different models (i.e., linear, non-linear, single best-SNP) in different cross-validation schemes, suggesting that perhaps the most promising approach for predicting expression variation from genotype would make some combination of models.

Analysis of the genomic features that both our extended KNN algorithm and the regularized linear model selected as important for prioritizing SNPs provides insight into specific classes of SNPs that may have better predictive power and offers concrete means by which we can efficiently explore the vast space of sequence variation and focus on the more relevant variation. Features that we found to have higher predictive power include proximity to the transcription start and end sites, high regional G/C content, and presence within microRNA or transcription factor binding sites. In contrast, we found that SNPs that are located far away from the gene and SNPs located within heterochromatic regions or low G/C content regions have, on average, lower predictive power. We also found that genes that are successfully predicted by our algorithm have, on average, higher expression and more variability and are enriched for classes of genes that have known functional roles in the measured cell types. This finding provides insight into the types of genes that we may expect to be able to predict in a given cell type.

There are many ways in which our algorithms can be further improved, including incorporating *trans* genetic variation, additional functional annotations of SNPs, and other types of genetic variation such as insertions, deletions, and copy number variation. Examining our ability to predict gene expression in other tissues using the models learned in this work is an interesting avenue of research, given that it has been shown that eQTLs are shared across different tissues [16], and that the similarity between gene expression profiles across different tissues is dominated by heritable effects at the cis-SNPs [34]. Our ability to provide highly robust predictions suggests that the time may be ripe for working on such problems, and that the ability to accurately predict some of the gene expression patterns of individuals using only their DNA sequence may be within reach.

## Methods

### Data and Code Download

All data used in the different models and the code generating the predictions presented here are available for download in the following URL: http://genie.weizmann.ac.il/software/gen2exp/gen2exp.html

### Genotype Dataset

We obtained genotype data from the HapMap project Phase II [8]. We removed the 60 children from the trios, resulting in 210 unrelated individuals from four distinct populations. We downloaded the entire set of measured single nucleotide polymorphisms (SNPs) for each individual, for a total of ∼3.5 M SNPs per individual. For each gene, we extracted all SNPs located inside the gene and those within 100 kb from the transcription start or end sites. We transformed each SNP to a discrete variable with values of 0, 1, or 2, corresponding to the number of minor alleles that each individual has for the SNP. Therefore, an individual with 0 minor alleles will have a value of 0, etc.

### Expression Dataset

We used expression measurements of 15,439 genes performed in lymphoblastoid cell lines for all HapMap individuals [10]. To remove population specific effects, we separately centered the expression of the gene (i.e., subtracted the mean to achieve zero mean) within each of the four populations.

### SNP Genomic Features Definition and Acquisition

The full list of SNP genomic features that we used along with their description and corresponding reference or website from which they were acquired are given in Table S15.

### Computing SNP Average Entropy per Gene

For each SNP we compute the entropy of its allele counts across all individuals based on Shannon's entropy calculation. Since different SNPs can have a different number of values (i.e., 0/1/2 vs. only 0/1), we first transform the minor allele count of each SNP into two alleles, A and B, such that 0 is converted to A = 0, B = 0, 1 to A = 0, B = 1 and 2 to A = 1, B = 1. Next, we compute Shannon's entropy for each of the alleles A and B, and average their result per SNP. For a specific gene, we average across all its cis-SNPs to obtain an average SNP entropy value per gene.

### Predictive Models

#### Single best–SNP model

For each gene, we selected the SNP with the highest correlation to expression in the training set. Using this SNP, we then constructed a predictor using simple linear regression between its minor allele counts and the expression of the corresponding gene. We computed the performance on the test set by predicting the expression values of unseen individuals using the resulting predictor.

#### Linear model with elastic-net regularization

For each gene, we used the training data (including expression and all cis-SNPs) to construct a linear regression model from the allele counts of all SNPs to the value of expression. To regularize the model, we used elastic-net [27], where the value of lambda (the penalty coefficient) was learned using cross-validation on the training set (i.e., partitioning the training set to a learning and validation set) and selecting the value that gave best results on the validation set. The training R^2^ was calculated as the average R^2^ achieved using internal cross-validation on the training set.

#### K-nearest-neighbor algorithm

K-nearest-neighbor (KNN) predicts the response value of a given instance using the response values of *k* instances that are closest to it in the feature space under some metric [25]. Intuitively, if instances are close to one another in the feature space, we expect them to be close to one another in the response space. KNN was shown to be a useful approach for many learning tasks [26], and since its naïve form has a single parameter (*k*), overfitting does not typically occur. Since the predictions made by KNN depend on the identity of the closest neighbors, the metric plays a key role in the predictive power of the method.

#### Simple KNN algorithm

For each gene, we selected the value of K that maximizes the variability explained by a predictor based on the average expression value of the K nearest neighbors of each individual. The distance metric was the sum of the absolute differences in minor allele counts across all cis-SNPs of the gene.

#### Extended KNN algorithm that integrates genomic features

To extend the above basic algorithm to integrate genomic features, we convert the genotype matrix into a feature matrix *X* of size *N_total_*×*D* and the expression values to a response vector *y* of size *N_total_*×1, where *N_total_* is the number of individuals in the task, and *D* is the number of SNPs or features that will be used for the prediction task. In addition, we define *F* as a matrix of genomic features representing different properties of the features themselves, where for each of the *D* features we have *M* genomic features, resulting in a matrix of size *D*×*M*. Our goal is to predict *y*, or minimize the squared error between the predicted response *y*? and the actual response *y*. We assessed the results that our algorithm achieved on a held-out test set to evaluate its performance. To this end, we first partition the data into training and test sets of sizes *N_train_* and *N_test_* = *N_total_*−*N_train_*, respectively, and ran the algorithms only on the training sets.

The extended version learns the distance metric used by the KNN. Instead of the metric used by the basic algorithm, in which every SNP is given an equal weight, the extended version learns a weight for each genomic feature and then uses this vector to differentially weigh each SNP within the distance metric, as a weighted sum of its genomic feature values. A key aspect of this approach is that each genomic feature is shared across all SNPs (with a corresponding value for each one), such that changing its coefficient within the genomic feature vector affects all SNPs, thereby reducing overfitting. In addition, it enables usage of additional information about the features which cannot be represented as part of the original *X* matrix of SNP values.

#### Regression using a greedy search over the space of linear combinations of genomic features

To learn one joint metric that uses all *M* genomic features, we define a metric by using a linear combination of all genomic features, using a coefficient vector α. In this approach, the distance between two instances *a,b* is defined as follows:
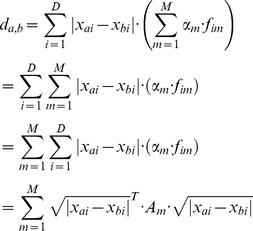
where 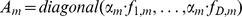
and *X_a,i_* is the value for the *i*'th feature of individual *a*, *f_i,m_* is the value of the *m*'th genomic feature for the feature *i*, and α*_m_* is the coefficient of the *m*'th genomic feature. Now, using this metric, we define the prediction for a single instance *n* as the average of its *k* nearest neighbors:
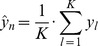



#### The loss function of the extended KNN algorithm

For the learning task, we can use several objective functions. In our case, since we are using a greedy heuristic approach, the objective function does not need to be differentiable. Although several objective functions are possible, we used the Metric-to-response Correlation (MRC) objective function, which is independent of *k*. During the optimization process of the metric, we attempt to maximize the correlation (across all instances) between the pairwise distances under the metric (*d_ab_*) to the corresponding distances in the response (|*y_a_−y_b_*|). Note that this objective function does not require *k*, and does not produce a prediction. Intuitively, it tries to learn the “right” metric, where for every two instances, the closer they are under the metric, the closer they are in their responses. Once we have optimized this objective, we can now select the *k* that will produce the prediction with the minimum L_2_ loss (i.e., maximum R^2^). The fact that the selection of *k* is detached from the metric learning can possibly help to reduce over-fitting and help the model to be more robust for different choices of *k*.

#### Optimization procedure for the extended KNN algorithm

To find the coefficient vector α that maximizes our selected objective function, we initialize a predictor pool with *M* predictors, where each predictor is based on one genomic feature, such that for predictor m: α_m_ = 1, α_j_ = 0 µ j≠m. We then compute the distances as described above, and for each predictor we compute the value of the objective function. From here on, we iterate the following algorithm:

1. Pick the *R*_max_ best predictors from the predictor pool in terms of their objective function, and define the objective function value for the *i*'th predictor as *f*_i_
2. For each pair of predictors *r_i_*, *r*_j_, *i* = 1 … *R*_max_, *j* = 1… *R*_max_, create the predictor *r*_ij_ in the following manner: for each *m* = 1… *M*:
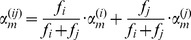

3. Define the new predictor pool (of size 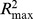) as all the predictors *r*_ij_, *i* = 1… *R*_max_, *j* = 1… *R*_max_

This way, every iteration tries to create better predictors by linearly combining two predictors from the previous pool. The combination is done in a weighted manner, where the relative weight of each predictor in the combination is the value of the objective function for this predictor (*f_i_*). This means that combining a “good” predictor with an “intermediate” predictor will result in a predictor more similar to the “good” one. Note that in every iteration each of the predictors in the predictor pool is a predictor with some vector of coefficients α over the *M* genomic features, which defines a metric. We stop iterating when we are not improving in the sense that the best *f_i_* is not increasing. Note that since we take all combinations of the top R_max_ predictors, we also get the combination of every predictor with itself, so the maximum value of *f_i_* in our predictor pool cannot decrease.

## Supporting Information

Figure S1 The multi-SNP algorithms exhibit better correspondence between predictions on the training and test set compared to a single best-SNP model. Shown are the training and test R^2^ values for all 15,439 genes, along with the Pearson and Spearman correlation coefficients between them. Results are shown for every cross validation scheme and for the KNN algorithm, linear regression model, combined model, and the model based on the SNP with the highest training set correlation.
(PDF)

Figure S2 PCA-corrected results. For each gene, the SNP genotype matrix of all individuals was corrected using the first 2 principal components, and the exact same analysis was conducted as in Figure 2 of the article. (A) Predictions of the KNN algorithm are more correlated between the training and test sets compared to the linear and best-SNP models. For the KNN-based algorithm, the regularized linear regression model, and the single best-SNP model that uses the most correlated SNP, shown are the Pearson and Spearman correlation coefficients between the training and test R^2^ across all genes and all three cross validation schemes. (B) For each of the three models and every cross-validation scheme, genes were binned by training R^2^. Shown is the mean training R^2^ of the genes in every bin. (C) For genes with the same training R^2^ in each model, the multi-SNP algorithms show higher test R^2^. For the same bins from (B), shown is the mean test R^2^ of the genes in every bin. (D) The combined algorithm is more robust than the all other models. For each model and every cross validation scheme, genes with training R^2^>0.05 were extracted. For each such set of genes, shown is the fraction (y-axis) of genes whose test R^2^ by each respective model and cross validation scheme were within some fraction (x-axis, robustness threshold) of their training R^2^.
(PDF)

Figure S3 Fraction of genes at various training R^2^ thresholds. For the set of 100, 200, 500, 1000, or 2000 genes with the largest variability in expression, shown is the fraction of genes (y-axis) whose training R^2^ is at least above some fraction (x-axis), for at least one cross validation scheme. Although the overall fraction of genes that are predicted with high training R^2^ is relatively small, our results indicate that these predictions are robust (i.e., their test R^2^ is close to their training R^2^).
(PDF)

Figure S4 The predictions of genes that are predicted well are generated using a smaller number of neighbors. For each cross validation scheme (for *Intra-Pop* results are shown separately for each population), shown is the distribution of the number of neighbors (value of *k* in the KNN algorithm) selected by the algorithm for genes that are predicted with test R^2^≥0.05 (blue) and genes predicted with test R^2^<0.05 (red).
(PDF)

Table S1 Number of genes that pass a certain R^2^ threshold in test/training using the different models in the Mixed-Pop cross-validation scheme.
(PDF)

Table S2 Number of genes that pass a certain R^2^ threshold in test/training using the different models in the Intra-Pop cross-validation scheme.
(PDF)

Table S3 Number of top predicted genes that overlap between different models at different cutoffs in the Mixed-Pop cross-validation scheme. KNN, K-Nearest-Neighbor; EN, Elastic-Net; SS, Single-SNP.
(PDF)

Table S4 Number of top predicted genes that overlap between different models at different cutoffs in the Intra-Pop cross-validation scheme. KNN, K-Nearest-Neighbor; EN, Elastic-Net; SS, Single-SNP.
(PDF)

Table S5 The list of the 100 top-predicted genes of the KNN model in the Cross-Pop cross-validation scheme.
(PDF)

Table S6 The list of the 100 top-predicted genes of the Elastic-Net model in the Cross-Pop cross-validation scheme.
(PDF)

Table S7 The list of the 100 top-predicted genes of the Single-SNP model in the Cross-Pop cross-validation scheme.
(PDF)

Table S8 The list of the 100 top-predicted genes of the KNN model in the Mixed-Pop cross-validation scheme.
(PDF)

Table S9 The list of the 100 top-predicted genes of the Elastic-Net model in the Mixed-Pop cross-validation scheme.
(PDF)

Table S10 The list of the 100 top-predicted genes of the Single-SNP model in the Mixed-Pop cross-validation scheme.
(PDF)

Table S11 The list of the 100 top-predicted genes of the KNN model in the Intra-Pop cross-validation scheme.
(PDF)

Table S12 The list of the 100 top-predicted genes of the Elastic-Net model in the Intra-Pop cross-validation scheme.
(PDF)

Table S13 The list of the 100 top-predicted genes of the Single-SNP model in the Intra-Pop cross-validation scheme.
(PDF)

Table S14 For each cross-validation scheme and model, shown is the percentage of genes (with test R∧2> = 0.05) where the model had the best predictive power.
(PDF)

Table S15 Full list of all genomic features complied for this paper.
(PDF)

Table S16 Enriched and depleted genomic features in multi-SNP models. Shown are SNP genomic features that were significantly enriched or depleted in both the extended-KNN algorithm and the regularized linear model. Enrichment/depletion of a genomic feature was defined as a feature with hypergeometric test p-value P<0.01 in all cross-validations.
(PDF)

Table S17 GO categories enrichment. Shown are the GO categories that are enriched in the set of 850 predictable genes. P-values were computed using Hypergeometric test and corrected using FDR with 5% threshold. Hypergeometric test and correction were performed by an in-house Perl script, and GO categories were downloaded from the gene ontology database (http://www.geneontology.org/).
(PDF)
