# Supplementary figures and images for: Robust Prediction of Expression Differences among Human Individuals Using Only Genotype Information
Source: PLoS Genet. 2013 Mar 28;9(3):e1003396. doi: 10.1371/journal.pgen.1003396 (PMC3610805; doi:10.1371/journal.pgen.1003396)

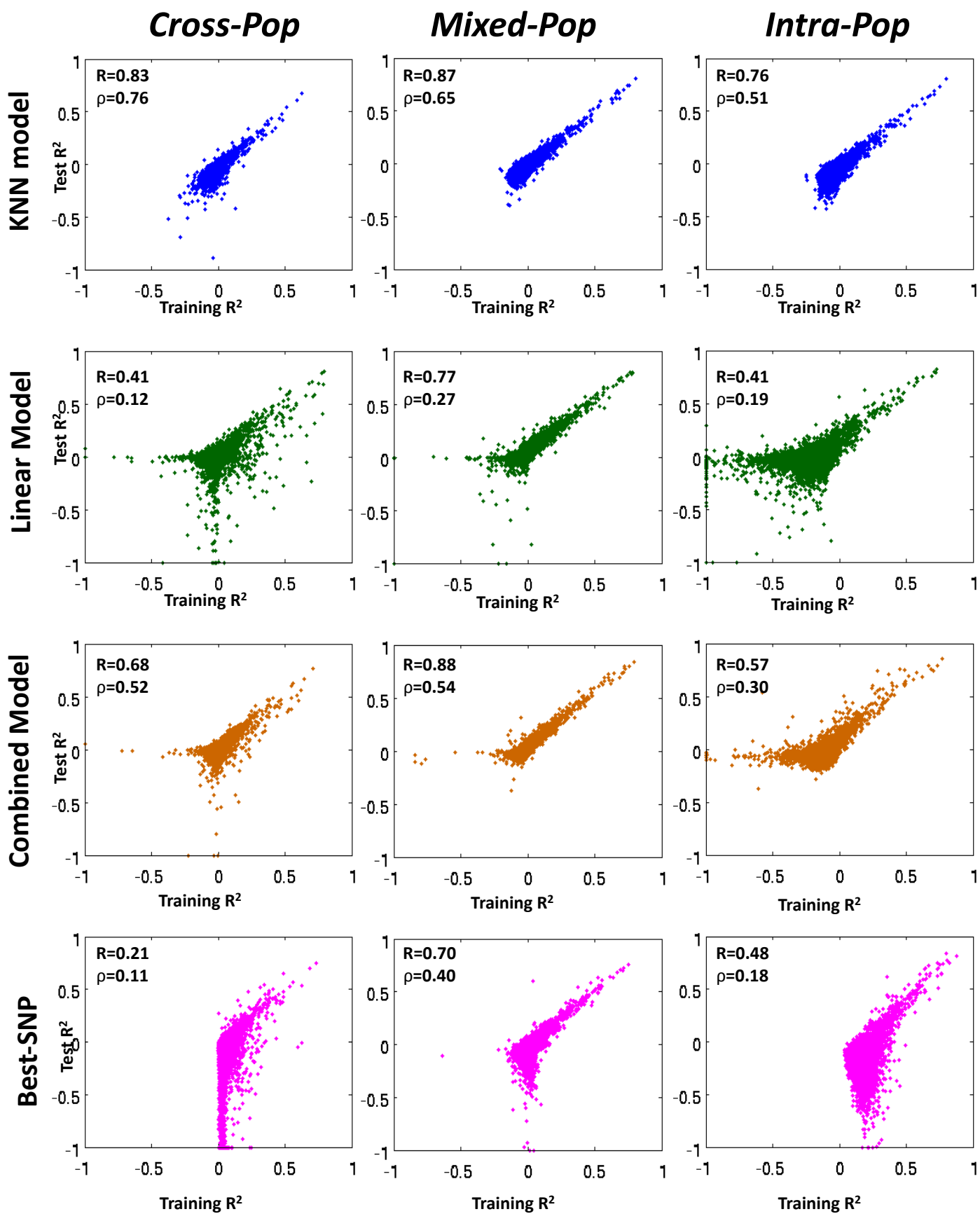

Supplement: Figure S1 — The multi-SNP algorithms exhibit better correspondence between predictions on the training and test set compared to a single best-SNP model. Shown are the training and test R2 values for all 15,439 genes, along with the Pearson and Spearman correlation coefficients between them. Results are shown for every cross validation scheme and for the KNN algorithm, linear regression model, combined model, and the model based on the SNP with the highest training set correlation. (PDF) [file pgen.1003396.s001.pdf]

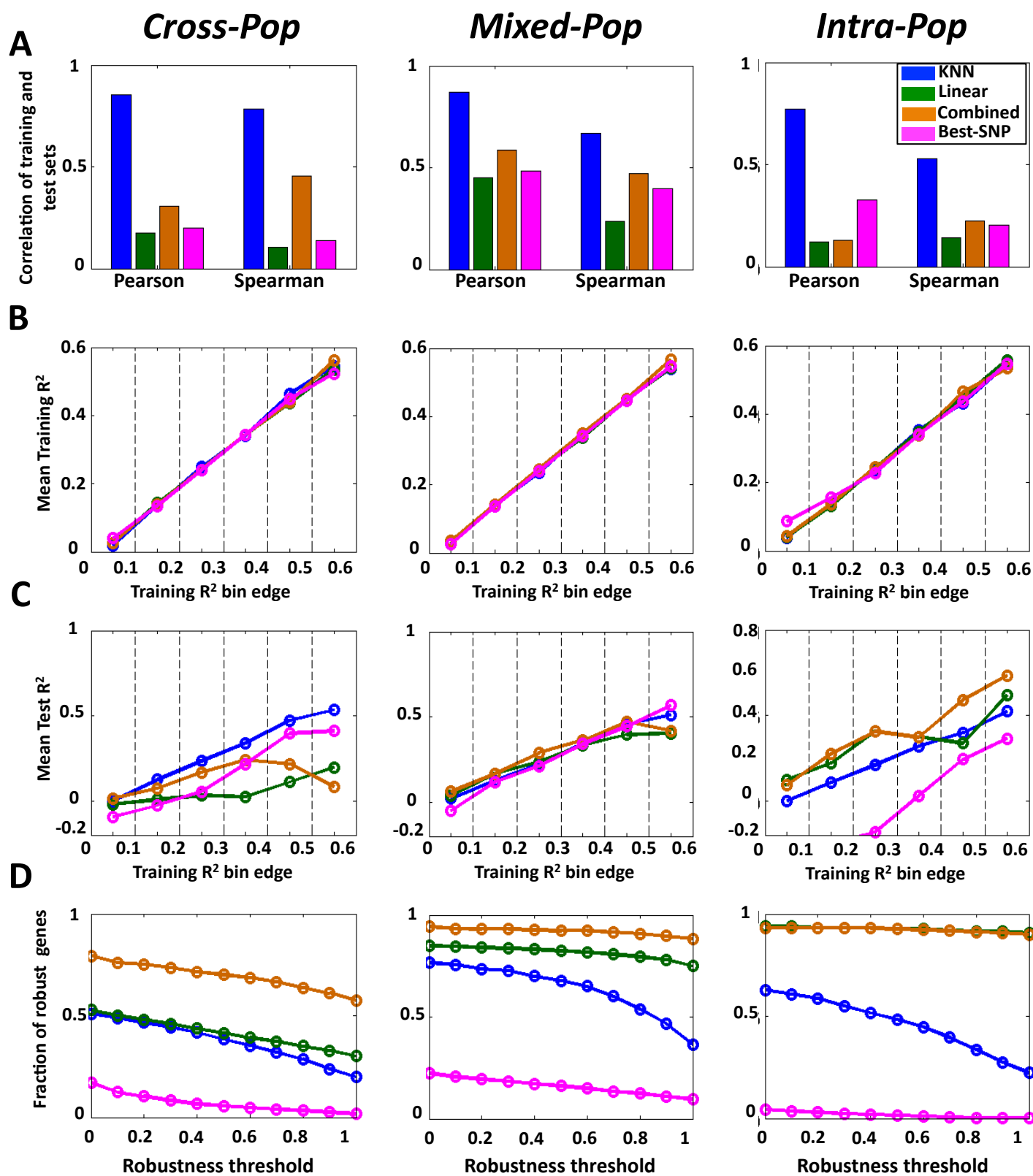

Supplement: Figure S2 — PCA-corrected results. For each gene, the SNP genotype matrix of all individuals was corrected using the first 2 principal components, and the exact same analysis was conducted as in Figure 2 of the article. (A) Predictions of the KNN algorithm are more correlated between the training and test sets compared to the linear and best-SNP models. For the KNN-based algorithm, the regularized linear regression model, and the single best-SNP model that uses the most correlated SNP, shown are the Pearson and Spearman correlation coefficients between the training and test R2 across all genes and all three cross validation schemes. (B) For each of the three models and every cross-validation scheme, genes were binned by training R2. Shown is the mean training R2 of the genes in every bin. (C) For genes with the same training R2 in each model, the multi-SNP algorithms show higher test R2. For the same bins from (B), shown is the mean test R2 of the genes in every bin. (D) The combined algorithm is more robust than the all other models. For each model and every cross validation scheme, genes with training R2>0.05 were extracted. For each such set of genes, shown is the fraction (y-axis) of genes whose test R2 by each respective model and cross validation scheme were within some fraction (x-axis, robustness threshold) of their training R2. (PDF) [file pgen.1003396.s002.pdf]

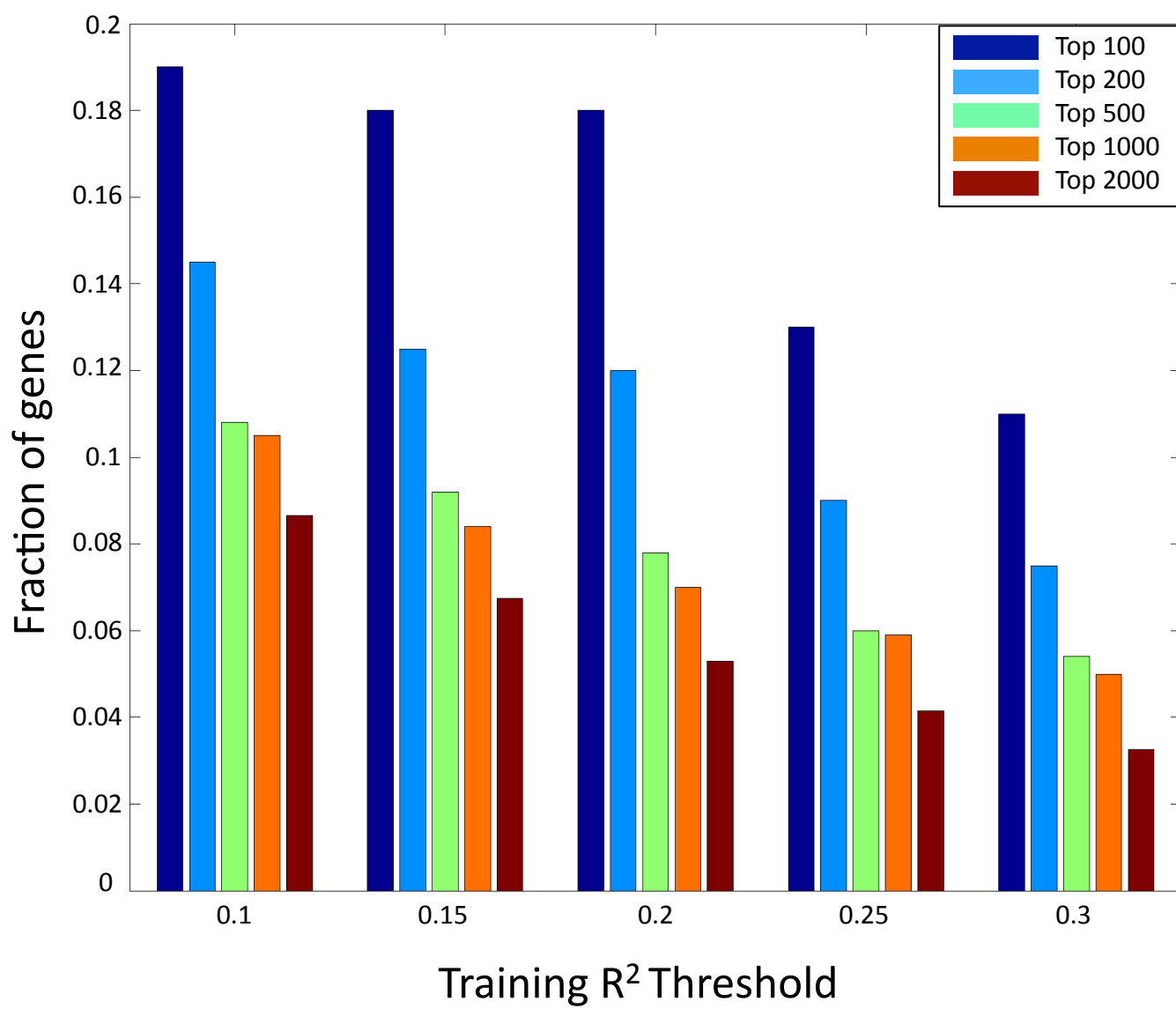

Supplement: Figure S3 — Fraction of genes at various training R2 thresholds. For the set of 100, 200, 500, 1000, or 2000 genes with the largest variability in expression, shown is the fraction of genes (y-axis) whose training R2 is at least above some fraction (x-axis), for at least one cross validation scheme. Although the overall fraction of genes that are predicted with high training R2 is relatively small, our results indicate that these predictions are robust (i.e., their test R2 is close to their training R2). (PDF) [file pgen.1003396.s003.pdf]

Cross-Pop  $P < 10^{-112}$

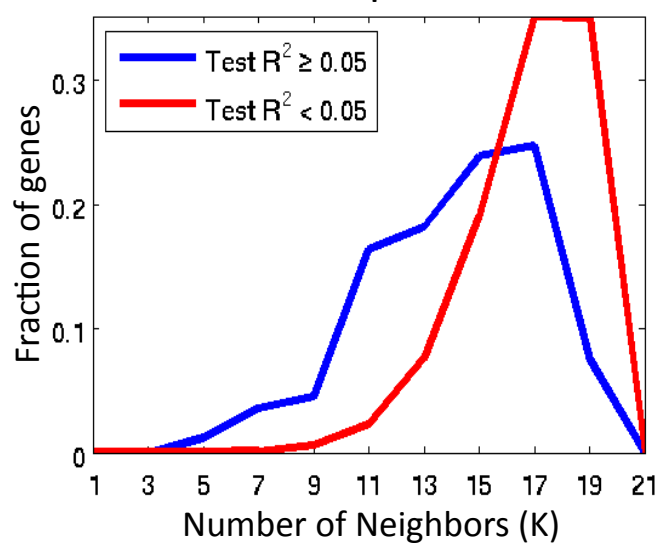

Mixed-Pop  $P < 10^{-200}$

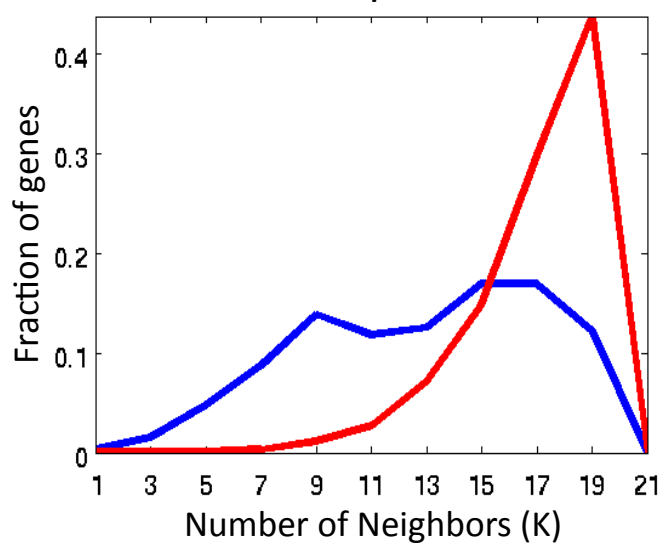

Intra-Pop (CHB)  $P < 10^{-259}$

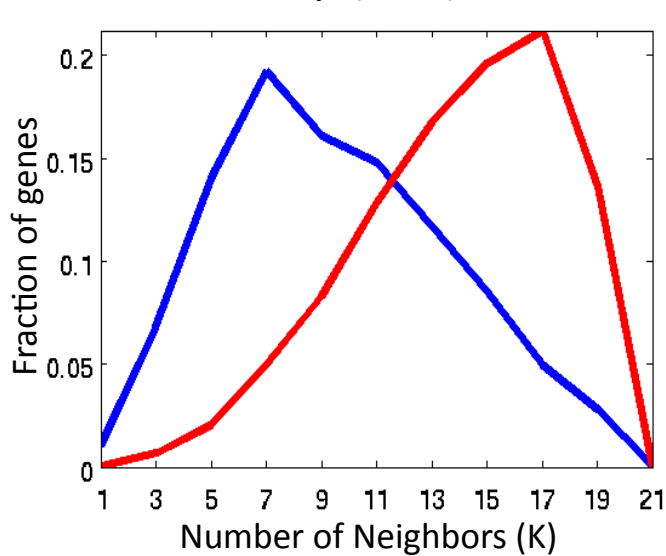

Intra-Pop (JPT)  $P < 10^{-274}$

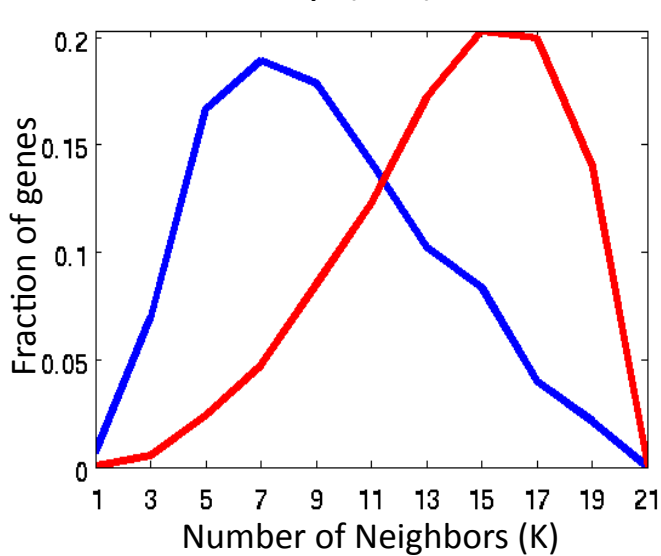

Intra-Pop (YRI)  $P < 10^{-225}$

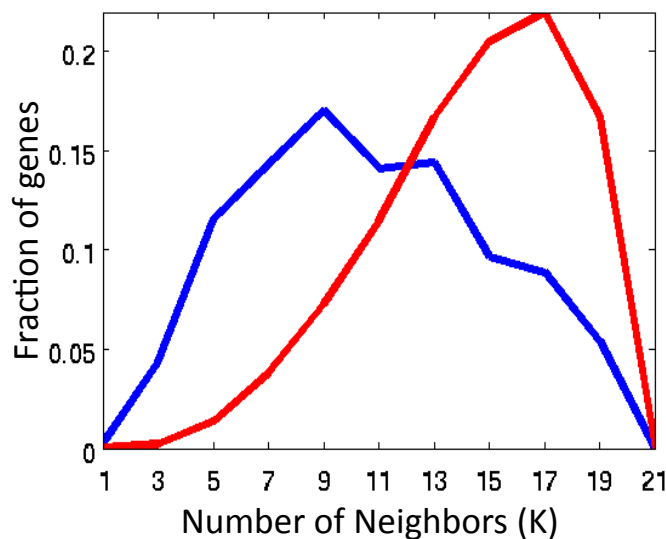

Intra-Pop (CEU)  $P < 10^{-274}$

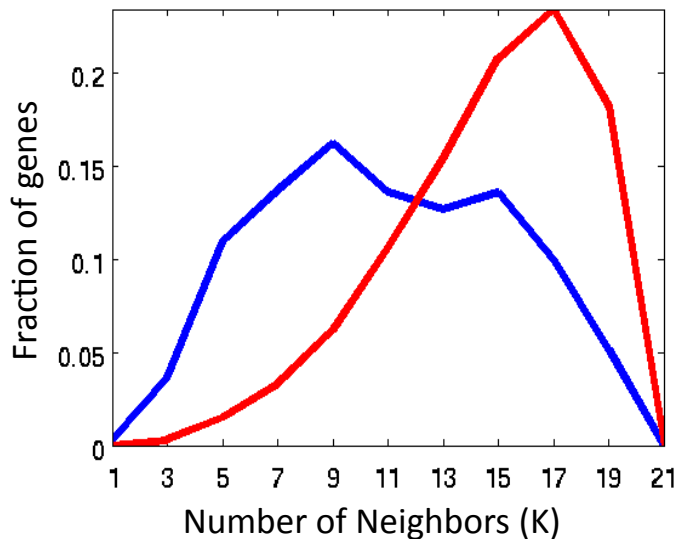

Supplement: Figure S4 — The predictions of genes that are predicted well are generated using a smaller number of neighbors. For each cross validation scheme (for Intra-Pop results are shown separately for each population), shown is the distribution of the number of neighbors (value of k in the KNN algorithm) selected by the algorithm for genes that are predicted with test R2≥0.05 (blue) and genes predicted with test R2<0.05 (red). (PDF) [file pgen.1003396.s004.pdf]
