# Supplementary material for: Robust Prediction of Expression Differences among Human Individuals Using Only Genotype Information
Source: PLoS Genet. 2013 Mar 28;9(3):e1003396. doi: 10.1371/journal.pgen.1003396 (PMC3610805; doi:10.1371/journal.pgen.1003396)
Supplement: Table S1 — Number of genes that pass a certain R2 threshold in test/training using the different models in the Mixed-Pop cross-validation scheme. (PDF) [file pgen.1003396.s005.pdf]

**Table S1. Number of genes that pass a certain $R^2$ threshold in test/training using the different models in the Mixed-Pop cross-validation scheme**

| R^2 threshold | KNN | Elastic-Net | Combined | Single SNP | All models |
|---|---|---|---|---|---|
| 0.05 | 503/627 | 559/567 | 555/558 | 829/1355 | 956/1419 |
| 0.1 | 278/340 | 356/359 | 338/341 | 448/575 | 529/624 |
| 0.2 | 103/129 | 171/173 | 149/151 | 172/199 | 214/234 |
| 0.3 | 56/62 | 85/86 | 69/69 | 81/86 | 103/109 |
| 0.4 | 28/32 | 49/50 | 38/38 | 43/46 | 57/61 |
| 0.5 | 18/18 | 26/28 | 19/19 | 17/19 | 30/32 |
