# Supplementary material for: Robust Prediction of Expression Differences among Human Individuals Using Only Genotype Information
Source: PLoS Genet. 2013 Mar 28;9(3):e1003396. doi: 10.1371/journal.pgen.1003396 (PMC3610805; doi:10.1371/journal.pgen.1003396)
Supplement: Table S2 — Number of genes that pass a certain R2 threshold in test/training using the different models in the Intra-Pop cross-validation scheme. (PDF) [file pgen.1003396.s006.pdf]

Table S2. Number of genes that pass a certain R^2 threshold in test/training using the different models in the Intra-Pop cross-validation scheme

R^2 threshol[illegible]	KNN	Elastic-Net	Combined	Single SNP	All models
0.05	320/488	160/161	226/227	524/15346	586/15348
0.1	190/283	105/106	143/143	382/14754	411/14756
0.2	76/101	59/59	70/70	203/2851	208/2852
0.3	37/46	39/39	40/40	118/472	119/473
0.4	19/24	22/22	19/19	65/150	67/150
0.5	12/14	11/11	9/9	34/67	40/68
