# Supplementary material for: Robust Prediction of Expression Differences among Human Individuals Using Only Genotype Information
Source: PLoS Genet. 2013 Mar 28;9(3):e1003396. doi: 10.1371/journal.pgen.1003396 (PMC3610805; doi:10.1371/journal.pgen.1003396)
Supplement: Table S3 — Number of top predicted genes that overlap between different models at different cutoffs in the Mixed-Pop cross-validation scheme. KNN, K-Nearest-Neighbor; EN, Elastic-Net; SS, Single-SNP. (PDF) [file pgen.1003396.s007.pdf]

Table S3. Number of top predicted genes that ovrelap between different models at different cutoffs in the Mixed-Pop cross-validation scheme. KNN, K-Nearest-Neighbor; EN, Elastic-Net; SS, Single-SNP.

Top genes	KNN-EN	KNN-SS	EN-SS	All models
10	7	5	6	4
20	14	11	12	9
50	36	30	37	26
100	74	64	72	58
200	143	131	149	117
