# Supplementary material for: Robust Prediction of Expression Differences among Human Individuals Using Only Genotype Information
Source: PLoS Genet. 2013 Mar 28;9(3):e1003396. doi: 10.1371/journal.pgen.1003396 (PMC3610805; doi:10.1371/journal.pgen.1003396)
Supplement: Table S4 — Number of top predicted genes that overlap between different models at different cutoffs in the Intra-Pop cross-validation scheme. KNN, K-Nearest-Neighbor; EN, Elastic-Net; SS, Single-SNP. (PDF) [file pgen.1003396.s008.pdf]

Table S4. Number of top predicted genes that overlap between different models at different cutoffs in the Intra-Pop cross-validation scheme. KNN, K-Nearest-Neighbor; EN, Elastic-Net; SS, Single-SNP.

# Top genes	KNN-EN	KNN-SS	EN-SS	All models
10	7	4	5	4
20	11	12	13	9
50	35	35	41	31
100	73	65	76	60
200	130	125	155	109
