# Supplementary material for: Robust Prediction of Expression Differences among Human Individuals Using Only Genotype Information
Source: PLoS Genet. 2013 Mar 28;9(3):e1003396. doi: 10.1371/journal.pgen.1003396 (PMC3610805; doi:10.1371/journal.pgen.1003396)
Supplement: Table S5 — The list of the 100 top-predicted genes of the KNN model in the Cross-Pop cross-validation scheme. (PDF) [file pgen.1003396.s009.pdf]

**Table S5. The list of the 100 top-predicted genes of the KNN model in the Cross-Pop cross-validation scheme**

| Entrez ID | Mean Test R^2 | Gene Symbol | Gene Description |
|---|---|---|---|
| 91612 | 0.67 | My015 | churchill domain containing 1 |
| 158160 | 0.56 | MGC88684 | hydroxysteroid (17-beta) dehydrogenase 7 pseudogene 2 |
| 84288 | 0.55 | RP11-290P14.1 | EF-hand calcium binding domain 2 |
| 93035 | 0.51 | PKHDL1 | polycystic kidney and hepatic disease 1 (autosomal recessive)-like 1 |
| 94101 | 0.45 | ORMDL1 | ORM1-like 1 (S. cerevisiae) |
| 6231 | 0.44 | RPS26 | ribosomal protein S26 |
| 829 | 0.41 | CAZ1 | capping protein (actin filament) muscle Z-line, alpha 1 |
| 3117 | 0.41 | HLA-DQA | major histocompatibility complex, class II, DQ alpha 1 |
| 3663 | 0.39 | IRF5 | interferon regulatory factor 5 |
| 91687 | 0.38 | RP3-383J4.1 | centromere protein L |
| 3127 | 0.38 | HLA-DRB5 | major histocompatibility complex, class II, DR beta 5 |
| 3123 | 0.37 | HLA-DRB1 | major histocompatibility complex, class II, DR beta 1 |
| 55556 | 0.33 | TYMSAS | enolase superfamily member 1 |
| 9045 | 0.33 | RPL14 | ribosomal protein L14 |
| 8904 | 0.32 | MGC1142 | copine I |
| 84833 | 0.31 | USMG5 | upregulated during skeletal muscle growth 5 homolog (mouse) |
| 81889 | 0.31 | YISKL | fumarylacetoacetate hydrolase domain containing 1 |
| 2635 | 0.31 | GBP3 | guanylate binding protein 3 |
| 115024 | 0.29 | NT5C3L | 5'-nucleotidase, cytosolic III-like |
| 55228 | 0.29 | FLJ10781 | hypothetical protein FLJ10781 |
| 858 | 0.29 | MGC12294 | caveolin 2 |
| 10494 | 0.28 | YSK1 | serine/threonine kinase 25 (STE20 homolog, yeast) |
| 282991 | 0.28 | RP11-316M21.4 | biogenesis of lysosome-related organelles complex-1, subunit 2 |
| 11118 | 0.27 | BTN3A2 | butyrophilin, subfamily 3, member A2 |
| 162394 | 0.26 | SLFN5 | schlafen family member 5 |
| 3652 | 0.25 | IPP | intracisternal A particle-promoted polypeptide |
| 87769 | 0.25 | RP11-151A6.2 | hypothetical protein BC004360 |
| 54535 | 0.25 | SBP | coiled-coil alpha-helical rod protein 1 |
| 7639 | 0.25 | ZNF85 | zinc finger protein 85 |
| 84617 | 0.25 | TUBB6 | tubulin, beta 6 |
| 255403 | 0.24 | ZNF718 | zinc finger protein 718 |
| 128272 | 0.24 | WGEF | Rho guanine nucleotide exchange factor (GEF) 19 |
| 7536 | 0.23 | ZNF162 | splicing factor 1 |
| 133383 | 0.23 | MGC33648 | hypothetical protein MGC33648 |
| 54014 | 0.23 | WDR9 | bromodomain and WD repeat domain containing 1 |
| 80817 | 0.22 | PS1TP3 | KIAA1712 |
| 23593 | 0.22 | SOUL | heme binding protein 2 |
| 219927 | 0.22 | MRPL21 | mitochondrial ribosomal protein L21 |
| 80008 | 0.22 | TMEM156 | transmembrane protein 156 |
| 51144 | 0.21 | KAR | hydroxysteroid (17-beta) dehydrogenase 12 |
| 113277 | 0.21 | TMEM106A | transmembrane protein 106A |
| 5411 | 0.21 | SDK3 | pinin, desmosome associated protein |
| 26519 | 0.21 | TIMM10 | translocase of inner mitochondrial membrane 10 homolog (yeast) |
| 3059 | 0.20 | HS1 | hematopoietic cell-specific Lyn substrate 1 |
| 152815 | 0.20 | THAP6 | THAP domain containing 6 |
| 6168 | 0.20 | RPL37A | ribosomal protein L37a |
| 25961 | 0.20 | NUDT13 | nudix (nucleoside diphosphate linked moiety X)-type motif 13 |
| 84314 | 0.20 | TMEM107 | transmembrane protein 107 |
| 84680 | 0.20 | PHACS | 1-aminocyclopropane-1-carboxylate synthase |
| 7311 | 0.20 | UBA52 | ubiquitin A-52 residue ribosomal protein fusion product 1 |
| 10953 | 0.20 | TOMM34 | translocase of outer mitochondrial membrane 34 |
| 29035 | 0.20 | PRO0149 | PRO0149 protein |
| 197322 | 0.19 | LOC197322 | hypothetical protein LOC197322 |
| 23008 | 0.19 | KIAA0265 | KIAA0265 protein |
| 130502 | 0.19 | LOC130502 | similar to CG14894-PA |
| 7390 | 0.19 | UROS | uroporphyrinogen III synthase (congenital erythropoietic porphyria) |
| 84516 | 0.19 | p25 | dynactin 5 (p25) |
| 10694 | 0.19 | KIAA0002 | chaperonin containing TCP1, subunit 8 (theta) |
| 2768 | 0.19 | RMP | guanine nucleotide binding protein (G protein) alpha 12 |
| 27102 | 0.19 | KIAA1369 | eukaryotic translation initiation factor 2-alpha kinase 1 |
| 9179 | 0.19 | MU-ARP2 | adaptor-related protein complex 4, mu 1 subunit |
| 7760 | 0.18 | ZNF213 | zinc finger protein 213 |
| 348094 | 0.18 | MGC120307 | ankyrin repeat and death domain containing 1A |
| 283726 | 0.18 | DKFZp666G057 | hypothetical protein DKFZp666G057 |
| 261726 | 0.17 | TIPRL | TIP41, TOR signalling pathway regulator-like (S. cerevisiae) |
| 57602 | 0.17 | USP36 | ubiquitin specific peptidase 36 |

| | | | |
|---|---|---|---|
| 3105 | 0.17 | HLA-A | major histocompatibility complex, class I, A |
| 9794 | 0.17 | MAML1 | mastermind-like 1 (Drosophila) |
| 10412 | 0.17 | YR-29 | TGF beta-inducible nuclear protein 1 |
| 387338 | 0.17 | RP4-603I14.2 | NOL1/NOP2/Sun domain family, member 4 |
| 1965 | 0.16 | EIF2S1 | eukaryotic translation initiation factor 2, subunit 1 alpha, 35kDa |
| 51275 | 0.16 | FLJ39616 | chromosome 12 open reading frame 47 |
| 1912 | 0.16 | PHC2 | polyhomeotic-like 2 (Drosophila) |
| 4552 | 0.16 | MTRR | 5-methyltetrahydrofolate-homocysteine methyltransferase reductase |
| 51101 | 0.16 | CGI-62 | chromosome 8 open reading frame 70 |
| 10233 | 0.16 | LRRC23 | leucine rich repeat containing 23 |
| 5037 | 0.16 | RKIP | phosphatidylethanolamine binding protein 1 |
| 55755 | 0.16 | MCPH3 | CDK5 regulatory subunit associated protein 2 |
| 1520 | 0.16 | MGC3886 | cathepsin S |
| 8034 | 0.15 | SLC25A16 | solute carrier family 25 (mitochondrial carrier; Graves disease autoantigen), member 16 |
| 7084 | 0.15 | TK2 | thymidine kinase 2, mitochondrial |
| 10645 | 0.15 | MGC15254 | calcium/calmodulin-dependent protein kinase kinase 2, beta |
| 3134 | 0.15 | HLAF | major histocompatibility complex, class I, F |
| 8693 | 0.15 | GALNT4 | UDP-N-acetyl-alpha-D-galactosamine:polypeptide N-acetylgalactosaminyltransferase 4 (GalNAc-T4) |
| 23042 | 0.15 | LP8165 | KIAA0251 protein |
| 10520 | 0.15 | ZNFC25 | zinc finger protein 211 |
| 2653 | 0.15 | NKH | glycine cleavage system protein H (aminomethyl carrier) |
| 285464 | 0.15 | FLJ34443 | hypothetical protein FLJ34443 |
| 4925 | 0.15 | NUCB2 | nucleobindin 2 |
| 81491 | 0.15 | PSP24B | G protein-coupled receptor 63 |
| 92070 | 0.15 | MGC21675 | hypothetical protein MGC21675 |
| 127700 | 0.15 | OSCP1 | chromosome 1 open reading frame 102 |
| 55666 | 0.14 | NPLOC4 | nuclear protein localization 4 homolog (S. cerevisiae) |
| 151963 | 0.14 | C3orf59 | chromosome 3 open reading frame 59 |
| 4835 | 0.14 | QR2 | NAD(P)H dehydrogenase, quinone 2 |
| 90416 | 0.14 | MGC20481 | coiled-coil domain containing 32 |
| 953 | 0.14 | NTPDase-1 | ectonucleoside triphosphate diphosphohydrolase 1 |
| 439921 | 0.14 | TMAP1 | matrix-remodelling associated 7 |
| 286354 | 0.14 | NAG12 | chromosome 9 open reading frame 130 |
| 977 | 0.14 | TSPAN24 | CD151 molecule (Raph blood group) |
