# Supplementary material for: Robust Prediction of Expression Differences among Human Individuals Using Only Genotype Information
Source: PLoS Genet. 2013 Mar 28;9(3):e1003396. doi: 10.1371/journal.pgen.1003396 (PMC3610805; doi:10.1371/journal.pgen.1003396)
Supplement: Table S6 — The list of the 100 top-predicted genes of the Elastic-Net model in the Cross-Pop cross-validation scheme. (PDF) [file pgen.1003396.s010.pdf]

Table S6. The list of the 100 top-predicted genes of the Elastic-Net model in the Cross-Pop cross-validation scheme

Entrez ID	Mean Test R^2	Gene Symbol	Gene Description
91612	0.81	My015	churchill domain containing 1
84833	0.79	USMG5	upregulated during skeletal muscle growth 5 homolog (mouse)
158160	0.71	MGC88684	hydroxysteroid (17-beta) dehydrogenase 7 pseudogene 2
3117	0.70	HLA-DQA	major histocompatibility complex, class II, DQ alpha 1
439921	0.69	TMAP1	matrix-remodelling associated 7
7311	0.68	UBA52	ubiquitin A-52 residue ribosomal protein fusion product 1
55228	0.64	FLJ10781	hypothetical protein FLJ10781
25902	0.62	MTHFD1L	methylenetetrahydrofolate dehydrogenase (NADP+ dependent) 1-like
10494	0.60	YSK1	serine/threonine kinase 25 (STE20 homolog, yeast)
282991	0.59	RP11-316M21.4	biogenesis of lysosome-related organelles complex-1, subunit 2
3123	0.59	HLA-DRB1	major histocompatibility complex, class II, DR beta 1
3105	0.58	HLA-A	major histocompatibility complex, class I, A
27004	0.57	TNG2	T-cell leukemia/lymphoma 6
2635	0.55	GBP3	guanylate binding protein 3
55755	0.53	MCPH3	CDK5 regulatory subunit associated protein 2
84288	0.50	RP11-290P14.1	EF-hand calcium binding domain 2
829	0.50	CAZ1	capping protein (actin filament) muscle Z-line, alpha 1
84641	0.49	MGC117350	hippocampus abundant transcript-like 1
91687	0.48	RP3-383J4.1	centromere protein L
7536	0.48	ZNF162	splicing factor 1
858	0.48	MGC12294	caveolin 2
11118	0.47	BTN3A2	butyrophilin, subfamily 3, member A2
9623	0.47	TML1	T-cell leukemia/lymphoma 1B
84680	0.45	PHACS	1-aminocyclopropane-1-carboxylate synthase
80008	0.44	TMEM156	transmembrane protein 156
8312	0.44	MGC52315	axin 1
3118	0.44	HLA-DXA	major histocompatibility complex, class II, DQ alpha 2
80817	0.43	PS1TP3	KIAA1712
7773	0.42	ZNF230	zinc finger protein 230
26519	0.42	TIMM10	translocase of inner mitochondrial membrane 10 homolog (yeast)
8904	0.42	MGC1142	copine I
4725	0.42	NDUFS5	NADH dehydrogenase (ubiquinone) Fe-S protein 5, 15kDa (NADH-coenzyme Q reductase)
255403	0.41	ZNF718	zinc finger protein 718
219927	0.41	MRPL21	mitochondrial ribosomal protein L21
94101	0.41	ORMDL1	ORM1-like 1 (S. cerevisiae)
115024	0.40	NT5C3L	5'-nucleotidase, cytosolic III-like
27068	0.40	SID6-306	pyrophosphatase (inorganic) 2
349565	0.40	PNAT3	nicotinamide nucleotide adenylyltransferase 3
977	0.39	TSPAN24	CD151 molecule (Raph blood group)
10953	0.39	TOMM34	translocase of outer mitochondrial membrane 34
155368	0.39	WBSCR27	Williams Beuren syndrome chromosome region 27
152815	0.38	THAP6	THAP domain containing 6
4731	0.38	NDUFV3	NADH dehydrogenase (ubiquinone) flavoprotein 3, 10kDa
7639	0.37	ZNF85	zinc finger protein 85
10352	0.37	WARS2	tryptophanyl tRNA synthetase 2 (mitochondrial)
348094	0.36	MGC120307	ankyrin repeat and death domain containing 1A
10694	0.36	KIAA0002	chaperonin containing TCP1, subunit 8 (theta)
6168	0.36	RPL37A	ribosomal protein L37a
51097	0.36	SCCPDH	saccharopine dehydrogenase (putative)
55556	0.36	TYMSAS	enolase superfamily member 1
130502	0.35	LOC130502	similar to CG14894-PA
6434	0.35	TRA2B	splicing factor, arginine/serine-rich 10 (transformer 2 homolog, Drosophila)
7367	0.34	UGT2B17	UDP glucuronosyltransferase 2 family, polypeptide B17
54535	0.34	SBP	coiled-coil alpha-helical rod protein 1
3652	0.34	IPP	intracisternal A particle-promoted polypeptide
133383	0.33	MGC33648	hypothetical protein MGC33648
80221	0.33	FLJ20920	hypothetical protein FLJ20920
81889	0.33	YISKL	fumarylacetoacetate hydrolase domain containing 1
10320	0.33	ZNFN1A1	zinc finger protein, subfamily 1A, 1 (Ikaros)
2944	0.33	MU	glutathione S-transferase M1
79672	0.33	FN3KRP	fructosamine-3-kinase-related protein
84516	0.32	p25	dynactin 5 (p25)
93035	0.32	PKHDL1	polycystic kidney and hepatic disease 1 (autosomal recessive)-like 1
11116	0.31	FOP	FGFR1 oncogene partner

9172	0.31	TTNAP	myomesin (M-protein) 2, 165kDa
126272	0.31	FLJ38944	EID-2-like inhibitor of differentiation-3
54822	0.31	TRP-PLIK	transient receptor potential cation channel, subfamily M, member 7
9056	0.31	Y+LAT1	solute carrier family 7 (cationic amino acid transporter, y+ system), member 7
1497	0.31	PQLC4	cystinosis, nephropathic
131076	0.30	FLJ33273	coiled-coil domain containing 58
84284	0.30	RP4-659I19.2	chromosome 1 open reading frame 57
84617	0.30	TUBB6	tubulin, beta 6
85019	0.30	MGC138577	chromosome 18 open reading frame 45
10645	0.30	MGC15254	calcium/calmodulin-dependent protein kinase kinase 2, beta
55588	0.30	MED29	intersex-like (Drosophila)
63897	0.30	FLJ22087	amplified in breast cancer 1
128272	0.30	WGEF	Rho guanine nucleotide exchange factor (GEF) 19
147138	0.30	TMC8	transmembrane channel-like 8
79762	0.30	RP11-322F10.4	chromosome 1 open reading frame 115
87769	0.29	RP11-151A6.2	hypothetical protein BC004360
91647	0.29	MGC29736	ATP synthase mitochondrial F1 complex assembly factor 2
64858	0.29	SNM1B	DNA cross-link repair 1B (PSO2 homolog, S. cerevisiae)
1984	0.29	uORF	eukaryotic translation initiation factor 5A
400566	0.28	LOC400566	hypothetical gene supported by AK128660
54475	0.28	Nle	notchless homolog 1 (Drosophila)
1117	0.28	YKL39	chitinase 3-like 2
23593	0.28	SOUL	heme binding protein 2
283726	0.28	DKFZp666G057	hypothetical protein DKFZp666G057
27143	0.28	PALD	KIAA1274
113277	0.28	TMEM106A	transmembrane protein 106A
115004	0.28	MGC131892	chromosome 6 open reading frame 150
51122	0.27	MGC57611	COMM domain containing 2
55278	0.27	QRSL1	glutaminyl-tRNA synthase (glutamine-hydrolyzing)-like 1
10653	0.27	SPINT2	serine peptidase inhibitor, Kunitz type, 2
56927	0.27	MGC14393	G protein-coupled receptor 108
51144	0.27	KAR	hydroxysteroid (17-beta) dehydrogenase 12
127428	0.27	RP4-758J24.3	chromosome 1 open reading frame 83
57001	0.27	DC11	ACN9 homolog (S. cerevisiae)
10267	0.26	RAMP1	receptor (calcitonin) activity modifying protein 1
127700	0.26	OSCP1	chromosome 1 open reading frame 102
