# Supplementary material for: Robust Prediction of Expression Differences among Human Individuals Using Only Genotype Information
Source: PLoS Genet. 2013 Mar 28;9(3):e1003396. doi: 10.1371/journal.pgen.1003396 (PMC3610805; doi:10.1371/journal.pgen.1003396)
Supplement: Table S7 — The list of the 100 top-predicted genes of the Single-SNP model in the Cross-Pop cross-validation scheme. (PDF) [file pgen.1003396.s011.pdf]

**Table S7. The list of the 100 top-predicted genes of the Single-SNP model in the Cross-Pop cross-validation scheme**

| Entrez ID | Mean Test R^2 | Gene Symbol | Gene Description |
|---|---|---|---|
| 3117 | 0.75 | HLA-DQA | major histocompatibility complex, class II, DQ alpha 1 |
| 158160 | 0.70 | MGC88684 | hydroxysteroid (17-beta) dehydrogenase 7 pseudogene 2 |
| 27004 | 0.65 | TNG2 | T-cell leukemia/lymphoma 6 |
| 25902 | 0.57 | MTHFD1L | methylenetetrahydrofolate dehydrogenase (NADP+ dependent) 1-like |
| 6168 | 0.54 | RPL37A | ribosomal protein L37a |
| 439921 | 0.54 | TMAP1 | matrix-remodelling associated 7 |
| 91612 | 0.53 | My015 | churchill domain containing 1 |
| 4731 | 0.52 | NDUFV3 | NADH dehydrogenase (ubiquinone) flavoprotein 3, 10kDa |
| 282991 | 0.50 | RP11-316M21.4 | biogenesis of lysosome-related organelles complex-1, subunit 2 |
| 9623 | 0.49 | TML1 | T-cell leukemia/lymphoma 1B |
| 829 | 0.48 | CAZ1 | capping protein (actin filament) muscle Z-line, alpha 1 |
| 84833 | 0.48 | USMG5 | upregulated during skeletal muscle growth 5 homolog (mouse) |
| 91687 | 0.47 | RP3-383J4.1 | centromere protein L |
| 84641 | 0.46 | MGC117350 | hippocampus abundant transcript-like 1 |
| 10494 | 0.45 | YSK1 | serine/threonine kinase 25 (STE20 homolog, yeast) |
| 7773 | 0.45 | ZNF230 | zinc finger protein 230 |
| 55755 | 0.43 | MCPH3 | CDK5 regulatory subunit associated protein 2 |
| 84288 | 0.43 | RP11-290P14.1 | EF-hand calcium binding domain 2 |
| 4725 | 0.42 | NDUFS5 | NADH dehydrogenase (ubiquinone) Fe-S protein 5, 15kDa (NADH-coenzyme Q reductase) |
| 115024 | 0.41 | NT5C3L | 5'-nucleotidase, cytosolic III-like |
| 7639 | 0.41 | ZNF85 | zinc finger protein 85 |
| 80008 | 0.41 | TMEM156 | transmembrane protein 156 |
| 349565 | 0.41 | PNAT3 | nicotinamide nucleotide adenylyltransferase 3 |
| 151246 | 0.40 | TRIPIN | shugoshin-like 2 (S. pombe) |
| 219927 | 0.40 | MRPL21 | mitochondrial ribosomal protein L21 |
| 80817 | 0.40 | PS1TP3 | KIAA1712 |
| 27068 | 0.39 | SID6-306 | pyrophosphatase (inorganic) 2 |
| 858 | 0.39 | MGC12294 | caveolin 2 |
| 11118 | 0.39 | BTN3A2 | butyrophilin, subfamily 3, member A2 |
| 133383 | 0.39 | MGC33648 | hypothetical protein MGC33648 |
| 155368 | 0.39 | WBSCR27 | Williams Beuren syndrome chromosome region 27 |
| 91647 | 0.38 | MGC29736 | ATP synthase mitochondrial F1 complex assembly factor 2 |
| 2944 | 0.38 | MU | glutathione S-transferase M1 |
| 8904 | 0.38 | MGC1142 | copine I |
| 10694 | 0.37 | KIAA0002 | chaperonin containing TCP1, subunit 8 (theta) |
| 7536 | 0.37 | ZNF162 | splicing factor 1 |
| 55228 | 0.37 | FLJ10781 | hypothetical protein FLJ10781 |
| 255403 | 0.37 | ZNF718 | zinc finger protein 718 |
| 2635 | 0.36 | GBP3 | guanylate binding protein 3 |
| 10320 | 0.36 | ZNFN1A1 | zinc finger protein, subfamily 1A, 1 (Ikaros) |
| 26519 | 0.36 | TIMM10 | translocase of inner mitochondrial membrane 10 homolog (yeast) |
| 8312 | 0.35 | MGC52315 | axin 1 |
| 84680 | 0.35 | PHACS | 1-aminocyclopropane-1-carboxylate synthase |
| 11116 | 0.34 | FOP | FGFR1 oncogene partner |
| 81889 | 0.34 | YISKL | fumarylacetoacetate hydrolase domain containing 1 |
| 93100 | 0.33 | PP3856 | nicotinate phosphoribosyltransferase domain containing 1 |
| 8878 | 0.33 | ZIP3 | sequestosome 1 |
| 130502 | 0.33 | LOC130502 | similar to CG14894-PA |
| 55871 | 0.33 | CBWD1 | COBW domain containing 1 |
| 10953 | 0.33 | TOMM34 | translocase of outer mitochondrial membrane 34 |
| 10267 | 0.32 | RAMP1 | receptor (calcitonin) activity modifying protein 1 |
| 131076 | 0.32 | FLJ33273 | coiled-coil domain containing 58 |
| 6434 | 0.32 | TRA2B | splicing factor, arginine/serine-rich 10 (transformer 2 homolog, Drosophila) |
| 147138 | 0.32 | TMC8 | transmembrane channel-like 8 |
| 55588 | 0.31 | MED29 | intersex-like (Drosophila) |
| 3118 | 0.31 | HLA-DXA | major histocompatibility complex, class II, DQ alpha 2 |
| 7311 | 0.31 | UBA52 | ubiquitin A-52 residue ribosomal protein fusion product 1 |
| 3652 | 0.31 | IPP | intracisternal A particle-promoted polypeptide |
| 51097 | 0.30 | SCCPDH | saccharopine dehydrogenase (putative) |
| 51144 | 0.30 | KAR | hydroxysteroid (17-beta) dehydrogenase 12 |
| 1497 | 0.30 | PQLC4 | cystinosis, nephropathic |
| 79672 | 0.29 | FN3KRP | fructosamine-3-kinase-related protein |
| 9172 | 0.29 | TTNAP | myomesin (M-protein) 2, 165kDa |
| 87769 | 0.28 | RP11-151A6.2 | hypothetical protein BC004360 |

| | | | |
|---|---|---|---|
| 197322 | 0.27 | LOC197322 | hypothetical protein LOC197322 |
| 54822 | 0.27 | TRP-PLIK | transient receptor potential cation channel, subfamily M, member 7 |
| 115004 | 0.27 | MGC131892 | chromosome 6 open reading frame 150 |
| 56927 | 0.27 | MGC14393 | G protein-coupled receptor 108 |
| 51122 | 0.27 | MGC57611 | COMM domain containing 2 |
| 55957 | 0.27 | MGC9505 | protein F25965 |
| 10653 | 0.26 | SPINT2 | serine peptidase inhibitor, Kunitz type, 2 |
| 3105 | 0.26 | HLA-A | major histocompatibility complex, class I, A |
| 128272 | 0.26 | WGEF | Rho guanine nucleotide exchange factor (GEF) 19 |
| 1984 | 0.26 | uORF | eukaryotic translation initiation factor 5A |
| 22900 | 0.26 | TUCAN | caspase recruitment domain family, member 8 |
| 84617 | 0.26 | TUBB6 | tubulin, beta 6 |
| 11014 | 0.25 | KDELR2 | KDEL (Lys-Asp-Glu-Leu) endoplasmic reticulum protein retention receptor 2 |
| 55556 | 0.25 | TYMSAS | enolase superfamily member 1 |
| 80221 | 0.25 | FLJ20920 | hypothetical protein FLJ20920 |
| 80315 | 0.25 | KIAA1673 | cytoplasmic polyadenylation element binding protein 4 |
| 1429 | 0.25 | FLJ41475 | crystallin, zeta (quinone reductase) |
| 93349 | 0.24 | MGC132667 | hypothetical protein BC004921 |
| 127428 | 0.24 | RP4-758J24.3 | chromosome 1 open reading frame 83 |
| 9794 | 0.24 | MAML1 | mastermind-like 1 (Drosophila) |
| 84284 | 0.24 | RP4-659I19.2 | chromosome 1 open reading frame 57 |
| 127700 | 0.24 | OSCP1 | chromosome 1 open reading frame 102 |
| 113277 | 0.24 | TMEM106A | transmembrane protein 106A |
| 64858 | 0.24 | SNM1B | DNA cross-link repair 1B (PSO2 homolog, S. cerevisiae) |
| 81544 | 0.24 | PP1665 | glycerophosphodiester phosphodiesterase domain containing 5 |
| 54475 | 0.23 | Nle | notchless homolog 1 (Drosophila) |
| 8310 | 0.23 | ACOX3 | acyl-Coenzyme A oxidase 3, pristanoyl |
| 1479 | 0.23 | MGC75122 | cleavage stimulation factor, 3' pre-RNA, subunit 3, 77kDa |
| 7390 | 0.23 | UROS | uroporphyrinogen III synthase (congenital erythropoietic porphyria) |
| 1776 | 0.23 | LSD | deoxyribonuclease I-like 3 |
| 10905 | 0.23 | MAN1B | mannosidase, alpha, class 1A, member 2 |
| 54535 | 0.23 | SBP | coiled-coil alpha-helical rod protein 1 |
| 147968 | 0.22 | MGC20576 | calpain 12 |
| 283726 | 0.22 | DKFZp666G057 | hypothetical protein DKFZp666G057 |
| 26284 | 0.22 | HERA-B | Era G-protein-like 1 (E. coli) |
| 1912 | 0.22 | PHC2 | polyhomeotic-like 2 (Drosophila) |
