# Supplementary material for: Robust Prediction of Expression Differences among Human Individuals Using Only Genotype Information
Source: PLoS Genet. 2013 Mar 28;9(3):e1003396. doi: 10.1371/journal.pgen.1003396 (PMC3610805; doi:10.1371/journal.pgen.1003396)
Supplement: Table S8 — The list of the 100 top-predicted genes of the KNN model in the Mixed-Pop cross-validation scheme. (PDF) [file pgen.1003396.s012.pdf]

Table S8. The list of the 100 top-predicted genes of the KNN model in the Mixed-Pop cross-validation scheme

Entrez ID	Mean Test R^2	Gene Symbol	Gene Description
91612	0.81	My015	churchill domain containing 1
6231	0.74	RPS26	ribosomal protein S26
94101	0.74	ORMDL1	ORM1-like 1 (S. cerevisiae)
93035	0.70	PKHDL1	polycystic kidney and hepatic disease 1 (autosomal recessive)-like 1
84641	0.70	MGC117350	hippocampus abundant transcript-like 1
7367	0.67	UGT2B17	UDP glucuronosyltransferase 2 family, polypeptide B17
7311	0.67	UBA52	ubiquitin A-52 residue ribosomal protein fusion product 1
84833	0.67	USMG5	upregulated during skeletal muscle growth 5 homolog (mouse)
282991	0.62	RP11-316M21.4	biogenesis of lysosome-related organelles complex-1, subunit 2
3123	0.62	HLA-DRB1	major histocompatibility complex, class II, DR beta 1
84288	0.62	RP11-290P14.1	EF-hand calcium binding domain 2
158160	0.62	MGC88684	hydroxysteroid (17-beta) dehydrogenase 7 pseudogene 2
9045	0.61	RPL14	ribosomal protein L14
3663	0.61	IRF5	interferon regulatory factor 5
858	0.59	MGC12294	caveolin 2
3105	0.58	HLA-A	major histocompatibility complex, class I, A
3127	0.55	HLA-DRB5	major histocompatibility complex, class II, DR beta 5
143570	0.53	XRRA1	X-ray radiation resistance associated 1
7536	0.52	ZNF162	splicing factor 1
3117	0.51	HLA-DQA	major histocompatibility complex, class II, DQ alpha 1
348094	0.47	MGC120307	ankyrin repeat and death domain containing 1A
829	0.45	CAZ1	capping protein (actin filament) muscle Z-line, alpha 1
4725	0.44	NDUFS5	NADH dehydrogenase (ubiquinone) Fe-S protein 5, 15kDa (NADH-coenzyme Q reductase)
54879	0.44	STLR	suppression of tumorigenicity 7 like
10494	0.43	YSK1	serine/threonine kinase 25 (STE20 homolog, yeast)
439921	0.42	TMAP1	matrix-remodelling associated 7
1965	0.41	EIF2S1	eukaryotic translation initiation factor 2, subunit 1 alpha, 35kDa
133383	0.40	MGC33648	hypothetical protein MGC33648
87769	0.40	RP11-151A6.2	hypothetical protein BC004360
91687	0.40	RP3-383J4.1	centromere protein L
8904	0.39	MGC1142	copine I
155368	0.39	WBSCR27	Williams Beuren syndrome chromosome region 27
23593	0.38	SOUL	heme binding protein 2
79618	0.38	PBHNF	homeobox containing 1
55556	0.37	TYMSAS	enolase superfamily member 1
84680	0.36	PHACS	1-aminocyclopropane-1-carboxylate synthase
10412	0.36	YR-29	TGF beta-inducible nuclear protein 1
219927	0.36	MRPL21	mitochondrial ribosomal protein L21
10233	0.35	LRRC23	leucine rich repeat containing 23
9419	0.35	HSPC139	postsynaptic protein CRIPT
93100	0.35	PP3856	nicotinate phosphoribosyltransferase domain containing 1
6199	0.34	STK14B	ribosomal protein S6 kinase, 70kDa, polypeptide 2
131076	0.34	FLJ33273	coiled-coil domain containing 58
255403	0.34	ZNF718	zinc finger protein 718
400566	0.34	LOC400566	hypothetical gene supported by AK128660
318	0.34	NUDT2	nudix (nucleoside diphosphate linked moiety X)-type motif 2
5411	0.34	SDK3	pinin, desmosome associated protein
10905	0.33	MAN1B	mannosidase, alpha, class 1A, member 2
162394	0.33	SLFN5	schlafen family member 5
11118	0.33	BTN3A2	butyrophilin, subfamily 3, member A2
2635	0.32	GBP3	guanylate binding protein 3
11147	0.32	HHLA3	HERV-H LTR-associating 3
5190	0.32	PXAAA1	peroxisomal biogenesis factor 6
6168	0.32	RPL37A	ribosomal protein L37a
84545	0.31	MRPL43	mitochondrial ribosomal protein L43
81889	0.31	YISKL	fumarylacetoacetate hydrolase domain containing 1
127255	0.31	MGC22773	leucine rich repeat containing 44
26519	0.31	TIMM10	translocase of inner mitochondrial membrane 10 homolog (yeast)
55755	0.31	MCPH3	CDK5 regulatory subunit associated protein 2
84314	0.30	TMEM107	transmembrane protein 107

3652	0.29	IPP	intracisternal A particle-promoted polypeptide
261726	0.29	TIPRL	TIP41, TOR signalling pathway regulator-like (S. cerevisiae)
197322	0.29	LOC197322	hypothetical protein LOC197322
115024	0.29	NT5C3L	5'-nucleotidase, cytosolic III-like
152815	0.28	THAP6	THAP domain containing 6
219736	0.28	STOX1	storkhead box 1
8034	0.28	SLC25A16	solute carrier family 25 (mitochondrial carrier; Graves disease autoantigen), member 16
3118	0.28	HLA-DXA	major histocompatibility complex, class II, DQ alpha 2
84221	0.27	MGC99490	chromosome 21 open reading frame 56
84617	0.27	TUBB6	tubulin, beta 6
55228	0.27	FLJ10781	hypothetical protein FLJ10781
146279	0.27	FLJ32871	hypothetical protein FLJ32871
55871	0.27	CBWD1	COBW domain containing 1
4552	0.26	MTRR	5-methyltetrahydrofolate-homocysteine methyltransferase reductase
51101	0.26	CGI-62	chromosome 8 open reading frame 70
63897	0.26	FLJ22087	amplified in breast cancer 1
84676	0.26	TRIM63	tripartite motif-containing 63
4835	0.26	QR2	NAD(P)H dehydrogenase, quinone 2
51074	0.26	MMRP19	APAF1 interacting protein
283726	0.25	DKFZp666G057	hypothetical protein DKFZp666G057
3059	0.25	HS1	hematopoietic cell-specific Lyn substrate 1
80008	0.25	TMEM156	transmembrane protein 156
57602	0.25	USP36	ubiquitin specific peptidase 36
51097	0.25	SCCPDH	saccharopine dehydrogenase (putative)
54535	0.25	SBP	coiled-coil alpha-helical rod protein 1
55278	0.24	QRSL1	glutaminyl-tRNA synthase (glutamine-hydrolyzing)-like 1
1635	0.24	MGC111062	dCMP deaminase
1912	0.24	PHC2	polyhomeotic-like 2 (Drosophila)
64858	0.23	SNM1B	DNA cross-link repair 1B (PSO2 homolog, S. cerevisiae)
10694	0.23	KIAA0002	chaperonin containing TCP1, subunit 8 (theta)
23008	0.23	KIAA0265	KIAA0265 protein
2683	0.23	MGC50983	UDP-Gal:betaGlcNAc beta 1,4- galactosyltransferase, polypeptide 1
8312	0.23	MGC52315	axin 1
7639	0.23	ZNF85	zinc finger protein 85
2021	0.23	FLJ27463	endonuclease G
7084	0.22	TK2	thymidine kinase 2, mitochondrial
10438	0.22	SUNCOR	nuclear DNA-binding protein
953	0.22	NTPDase-1	ectonucleoside triphosphate diphosphohydrolase 1
10352	0.22	WARS2	tryptophanyl tRNA synthetase 2 (mitochondrial)
915	0.22	T3D	CD3d molecule, delta (CD3-TCR complex)
