# Supplementary material for: Robust Prediction of Expression Differences among Human Individuals Using Only Genotype Information
Source: PLoS Genet. 2013 Mar 28;9(3):e1003396. doi: 10.1371/journal.pgen.1003396 (PMC3610805; doi:10.1371/journal.pgen.1003396)
Supplement: Table S9 — The list of the 100 top-predicted genes of the Elastic-Net model in the Mixed-Pop cross-validation scheme. (PDF) [file pgen.1003396.s013.pdf]

Table S9. The list of the 100 top-predicted genes of the Elastic-Net model in the Mixed-Pop cross-validation scheme

Entrez ID	Mean Test R^2	Gene Symbol	Gene Description
91612	0.80	My015	churchill domain containing 1
94101	0.80	ORMDL1	ORM1-like 1 (S. cerevisiae)
7311	0.79	UBA52	ubiquitin A-52 residue ribosomal protein fusion product 1
84833	0.79	USMG5	upregulated during skeletal muscle growth 5 homolog (mouse)
3123	0.78	HLA-DRB1	major histocompatibility complex, class II, DR beta 1
3117	0.77	HLA-DQA	major histocompatibility complex, class II, DQ alpha 1
6231	0.75	RPS26	ribosomal protein S26
439921	0.74	TMAP1	matrix-remodelling associated 7
27004	0.73	TNG2	T-cell leukemia/lymphoma 6
93035	0.73	PKHDL1	polycystic kidney and hepatic disease 1 (autosomal recessive)-like 1
158160	0.72	MGC88684	hydroxysteroid (17-beta) dehydrogenase 7 pseudogene 2
3105	0.68	HLA-A	major histocompatibility complex, class I, A
3663	0.67	IRF5	interferon regulatory factor 5
400566	0.66	LOC400566	hypothetical gene supported by AK128660
84288	0.65	RP11-290P14.1	EF-hand calcium binding domain 2
84680	0.65	PHACS	1-aminocyclopropane-1-carboxylate synthase
3127	0.61	HLA-DRB5	major histocompatibility complex, class II, DR beta 5
2635	0.60	GBP3	guanylate binding protein 3
3118	0.59	HLA-DXA	major histocompatibility complex, class II, DQ alpha 2
858	0.59	MGC12294	caveolin 2
7536	0.58	ZNF162	splicing factor 1
153733	0.57	MGC39633	coiled-coil domain containing 112
9045	0.56	RPL14	ribosomal protein L14
282991	0.56	RP11-316M21.4	biogenesis of lysosome-related organelles complex-1, subunit 2
10494	0.56	YSK1	serine/threonine kinase 25 (STE20 homolog, yeast)
93100	0.55	PP3856	nicotinate phosphoribosyltransferase domain containing 1
6168	0.55	RPL37A	ribosomal protein L37a
4731	0.55	NDUFV3	NADH dehydrogenase (ubiquinone) flavoprotein 3, 10kDa
2944	0.52	MU	glutathione S-transferase M1
55228	0.52	FLJ10781	hypothetical protein FLJ10781
9623	0.51	TML1	T-cell leukemia/lymphoma 1B
87769	0.51	RP11-151A6.2	hypothetical protein BC004360
348094	0.50	MGC120307	ankyrin repeat and death domain containing 1A
197322	0.50	LOC197322	hypothetical protein LOC197322
55755	0.50	MCPH3	CDK5 regulatory subunit associated protein 2
255403	0.50	ZNF718	zinc finger protein 718
143570	0.50	XRRA1	X-ray radiation resistance associated 1
79618	0.49	PBHNF	homeobox containing 1
829	0.49	CAZ1	capping protein (actin filament) muscle Z-line, alpha 1
11118	0.49	BTN3A2	butyrophilin, subfamily 3, member A2
84641	0.48	MGC117350	hippocampus abundant transcript-like 1
8312	0.48	MGC52315	axin 1
131076	0.48	FLJ33273	coiled-coil domain containing 58
55556	0.47	TYMSAS	enolase superfamily member 1
349565	0.47	PNAT3	nicotinamide nucleotide adenylyltransferase 3
25902	0.47	MTHFD1L	methylenetetrahydrofolate dehydrogenase (NADP+ dependent) 1-like
6199	0.46	STK14B	ribosomal protein S6 kinase, 70kDa, polypeptide 2
91687	0.46	RP3-383J4.1	centromere protein L
54879	0.46	STLR	suppression of tumorigenicity 7 like
23593	0.45	SOUL	heme binding protein 2
155368	0.44	WBSCR27	Williams Beuren syndrome chromosome region 27
4725	0.44	NDUFS5	NADH dehydrogenase (ubiquinone) Fe-S protein 5, 15kDa (NADH-coenzyme Q reductase)
7367	0.44	UGT2B17	UDP glucuronosyltransferase 2 family, polypeptide B17
133383	0.43	MGC33648	hypothetical protein MGC33648
27068	0.43	SID6-306	pyrophosphatase (inorganic) 2
10438	0.43	SUNCOR	nuclear DNA-binding protein
7773	0.42	ZNF230	zinc finger protein 230
219927	0.42	MRPL21	mitochondrial ribosomal protein L21
283726	0.42	DKFZp666G057	hypothetical protein DKFZp666G057

1965	0.41	EIF2S1	eukaryotic translation initiation factor 2, subunit 1 alpha, 35kDa
51097	0.41	SCCPDH	saccharopine dehydrogenase (putative)
10352	0.41	WARS2	tryptophanyl tRNA synthetase 2 (mitochondrial)
10694	0.40	KIAA0002	chaperonin containing TCP1, subunit 8 (theta)
162394	0.40	SLFN5	schlafen family member 5
80817	0.40	PS1TP3	KIAA1712
11147	0.40	HHLA3	HERV-H LTR-associating 3
55278	0.40	QRSL1	glutaminyl-tRNA synthase (glutamine-hydrolyzing)-like 1
318	0.40	NUDT2	nudix (nucleoside diphosphate linked moiety X)-type motif 2
84221	0.39	MGC99490	chromosome 21 open reading frame 56
80221	0.39	FLJ20920	hypothetical protein FLJ20920
10905	0.39	MAN1B	mannosidase, alpha, class 1A, member 2
5190	0.39	PXAAA1	peroxisomal biogenesis factor 6
261726	0.39	TIPRL	TIP41, TOR signalling pathway regulator-like (S. cerevisiae)
8904	0.39	MGC1142	copine I
8605	0.37	PLA2G4C	phospholipase A2, group IVC (cytosolic, calcium-independent)
80008	0.37	TMEM156	transmembrane protein 156
115024	0.37	NT5C3L	5'-nucleotidase, cytosolic III-like
10953	0.37	TOMM34	translocase of outer mitochondrial membrane 34
54535	0.37	SBP	coiled-coil alpha-helical rod protein 1
5434	0.37	XAP4	polymerase (RNA) II (DNA directed) polypeptide E, 25kDa
10077	0.37	TSSC6	tetraspanin 32
9317	0.37	RPR-1	phosphotriesterase related
11116	0.37	FOP	FGFR1 oncogene partner
55871	0.36	CBWD1	COBW domain containing 1
1117	0.36	YKL39	chitinase 3-like 2
64858	0.36	SNM1B	DNA cross-link repair 1B (PSO2 homolog, S. cerevisiae)
10233	0.36	LRRC23	leucine rich repeat containing 23
10267	0.36	RAMP1	receptor (calcitonin) activity modifying protein 1
26519	0.35	TIMM10	translocase of inner mitochondrial membrane 10 homolog (yeast)
10645	0.35	MGC15254	calcium/calmodulin-dependent protein kinase kinase 2, beta
10320	0.35	ZNFN1A1	zinc finger protein, subfamily 1A, 1 (Ikaros)
1912	0.35	PHC2	polyhomeotic-like 2 (Drosophila)
7639	0.35	ZNF85	zinc finger protein 85
79672	0.35	FN3KRP	fructosamine-3-kinase-related protein
339231	0.34	ARL16	ADP-ribosylation factor-like 16
51074	0.34	MMRP19	APAF1 interacting protein
126272	0.34	FLJ38944	EID-2-like inhibitor of differentiation-3
22927	0.34	SERBP1L	hyaluronan binding protein 4
25885	0.34	RPO1-4	polymerase (RNA) I polypeptide A, 194kDa
84314	0.34	TMEM107	transmembrane protein 107
