# Supplementary material for: Robust Prediction of Expression Differences among Human Individuals Using Only Genotype Information
Source: PLoS Genet. 2013 Mar 28;9(3):e1003396. doi: 10.1371/journal.pgen.1003396 (PMC3610805; doi:10.1371/journal.pgen.1003396)
Supplement: Table S10 — The list of the 100 top-predicted genes of the Single-SNP model in the Mixed-Pop cross-validation scheme. (PDF) [file pgen.1003396.s014.pdf]

Table S10. The list of the 100 top-predicted genes of the Single-SNP model in the Mixed-Pop cross-validation scheme

Entrez ID	Mean Test R^2	Gene Symbol	Gene Description
3117	0.75	HLA-DQA	major histocompatibility complex, class II, DQ alpha 1
91612	0.72	My015	churchill domain containing 1
27004	0.71	TNG2	T-cell leukemia/lymphoma 6
158160	0.70	MGC88684	hydroxysteroid (17-beta) dehydrogenase 7 pseudogene 2
84833	0.69	USMG5	upregulated during skeletal muscle growth 5 homolog (mouse)
7311	0.64	UBA52	ubiquitin A-52 residue ribosomal protein fusion product 1
93035	0.63	PKHDL1	polycystic kidney and hepatic disease 1 (autosomal recessive)-like 1
282991	0.63	RP11-316M21.4	biogenesis of lysosome-related organelles complex-1, subunit 2
6168	0.61	RPL37A	ribosomal protein L37a
83543	0.60	MGC29466	chromosome 9 open reading frame 58
25902	0.59	MTHFD1L	methylenetetrahydrofolate dehydrogenase (NADP+ dependent) 1-like
439921	0.56	TMAP1	matrix-remodelling associated 7
4731	0.56	NDUFV3	NADH dehydrogenase (ubiquinone) flavoprotein 3, 10kDa
94101	0.55	ORMDL1	ORM1-like 1 (S. cerevisiae)
84288	0.55	RP11-290P14.1	EF-hand calcium binding domain 2
84641	0.54	MGC117350	hippocampus abundant transcript-like 1
10494	0.54	YSK1	serine/threonine kinase 25 (STE20 homolog, yeast)
9623	0.53	TML1	T-cell leukemia/lymphoma 1B
84680	0.53	PHACS	1-aminocyclopropane-1-carboxylate synthase
858	0.52	MGC12294	caveolin 2
6231	0.51	RPS26	ribosomal protein S26
91687	0.51	RP3-383J4.1	centromere protein L
829	0.51	CAZ1	capping protein (actin filament) muscle Z-line, alpha 1
3663	0.50	IRF5	interferon regulatory factor 5
255403	0.49	ZNF718	zinc finger protein 718
55755	0.48	MCPH3	CDK5 regulatory subunit associated protein 2
348094	0.47	MGC120307	ankyrin repeat and death domain containing 1A
6199	0.47	STK14B	ribosomal protein S6 kinase, 70kDa, polypeptide 2
219927	0.45	MRPL21	mitochondrial ribosomal protein L21
87769	0.45	RP11-151A6.2	hypothetical protein BC004360
133383	0.45	MGC33648	hypothetical protein MGC33648
8312	0.45	MGC52315	axin 1
155368	0.44	WBSCR27	Williams Beuren syndrome chromosome region 27
3105	0.44	HLA-A	major histocompatibility complex, class I, A
7773	0.44	ZNF230	zinc finger protein 230
2635	0.44	GBP3	guanylate binding protein 3
2944	0.44	MU	glutathione S-transferase M1
55228	0.43	FLJ10781	hypothetical protein FLJ10781
80008	0.43	TMEM156	transmembrane protein 156
7536	0.42	ZNF162	splicing factor 1
27068	0.42	SID6-306	pyrophosphatase (inorganic) 2
3118	0.42	HLA-DXA	major histocompatibility complex, class II, DQ alpha 2
131076	0.42	FLJ33273	coiled-coil domain containing 58
151246	0.42	TRIPIN	shugoshin-like 2 (S. pombe)
9045	0.42	RPL14	ribosomal protein L14
115024	0.41	NT5C3L	5'-nucleotidase, cytosolic III-like
283726	0.41	DKFZp666G057	hypothetical protein DKFZp666G057
4725	0.41	NDUFS5	NADH dehydrogenase (ubiquinone) Fe-S protein 5, 15kDa (NADH-coenzyme Q reductase)
80817	0.41	PS1TP3	KIAA1712
7639	0.41	ZNF85	zinc finger protein 85
10953	0.40	TOMM34	translocase of outer mitochondrial membrane 34
11118	0.40	BTN3A2	butyrophilin, subfamily 3, member A2
197322	0.40	LOC197322	hypothetical protein LOC197322
349565	0.40	PNAT3	nicotinamide nucleotide adenylyltransferase 3
11147	0.39	HHLA3	HERV-H LTR-associating 3
10694	0.39	KIAA0002	chaperonin containing TCP1, subunit 8 (theta)
91647	0.39	MGC29736	ATP synthase mitochondrial F1 complex assembly factor 2
10905	0.38	MAN1B	mannosidase, alpha, class 1A, member 2
26519	0.37	TIMM10	translocase of inner mitochondrial membrane 10 homolog (yeast)

8904	0.37	MGC1142	copine I
55556	0.37	TYMSAS	enolase superfamily member 1
10320	0.37	ZNFN1A1	zinc finger protein, subfamily 1A, 1 (Ikaros)
93100	0.37	PP3856	nicotinate phosphoribosyltransferase domain containing 1
80221	0.37	FLJ20920	hypothetical protein FLJ20920
8878	0.37	ZIP3	sequestosome 1
55871	0.36	CBWD1	COBW domain containing 1
84676	0.35	TRIM63	tripartite motif-containing 63
10412	0.35	YR-29	TGF beta-inducible nuclear protein 1
10233	0.35	LRRC23	leucine rich repeat containing 23
64858	0.35	SNM1B	DNA cross-link repair 1B (PSO2 homolog, S. cerevisiae)
9172	0.34	TTNAP	myomesin (M-protein) 2, 165kDa
3123	0.34	HLA-DRB1	major histocompatibility complex, class II, DR beta 1
11116	0.34	FOP	FGFR1 oncogene partner
389362	0.34	RP11-506K6.3	hypothetical LOC389362
6434	0.33	TRA2B	splicing factor, arginine/serine-rich 10 (transformer 2 homolog, Drosophila)
54465	0.33	FLJ22647	ETAA16
130502	0.33	LOC130502	similar to CG14894-PA
147138	0.33	TMC8	transmembrane channel-like 8
1965	0.33	EIF2S1	eukaryotic translation initiation factor 2, subunit 1 alpha, 35kDa
10267	0.32	RAMP1	receptor (calcitonin) activity modifying protein 1
220	0.32	RALDH3	aldehyde dehydrogenase 1 family, member A3
54535	0.32	SBP	coiled-coil alpha-helical rod protein 1
51101	0.32	CGI-62	chromosome 8 open reading frame 70
127428	0.32	RP4-758J24.3	chromosome 1 open reading frame 83
81889	0.31	YISKL	fumarylacetoacetate hydrolase domain containing 1
1497	0.31	PQLC4	cystinosis, nephropathic
3652	0.31	IPP	intracisternal A particle-promoted polypeptide
55278	0.31	QRSL1	glutaminyl-tRNA synthase (glutamine-hydrolyzing)-like 1
93349	0.31	MGC132667	hypothetical protein BC004921
51122	0.31	MGC57611	COMM domain containing 2
145173	0.31	Gal-T	beta 1,3-galactosyltransferase-like
80315	0.31	KIAA1673	cytoplasmic polyadenylation element binding protein 4
51097	0.30	SCCPDH	saccharopine dehydrogenase (putative)
22900	0.30	TUCAN	caspase recruitment domain family, member 8
51144	0.30	KAR	hydroxysteroid (17-beta) dehydrogenase 12
55588	0.30	MED29	intersex-like (Drosophila)
1479	0.30	MGC75122	cleavage stimulation factor, 3' pre-RNA, subunit 3, 77kDa
80007	0.30	FLJ13490	chromosome 10 open reading frame 88
10438	0.30	SUNCOR	nuclear DNA-binding protein
5411	0.29	SDK3	pinin, desmosome associated protein
