# Supplementary material for: Robust Prediction of Expression Differences among Human Individuals Using Only Genotype Information
Source: PLoS Genet. 2013 Mar 28;9(3):e1003396. doi: 10.1371/journal.pgen.1003396 (PMC3610805; doi:10.1371/journal.pgen.1003396)
Supplement: Table S11 — The list of the 100 top-predicted genes of the KNN model in the Intra-Pop cross-validation scheme. (PDF) [file pgen.1003396.s015.pdf]

Table S11. The list of the 100 top-predicted genes of the KNN model in the Intra-Pop cross-validation scheme

Entrez ID	Mean Test R^2	Gene Symbol	Gene Description
91612	0.81	My015	churchill domain containing 1
94101	0.73	ORMDL1	ORM1-like 1 (S. cerevisiae)
6231	0.69	RPS26	ribosomal protein S26
93035	0.66	PKHDL1	polycystic kidney and hepatic disease 1 (autosomal recessive)-like 1
7367	0.65	UGT2B17	UDP glucuronosyltransferase 2 family, polypeptide B17
158160	0.63	MGC88684	hydroxysteroid (17-beta) dehydrogenase 7 pseudogene 2
3663	0.62	IRF5	interferon regulatory factor 5
3123	0.57	HLA-DRB1	major histocompatibility complex, class II, DR beta 1
84288	0.55	RP11-290P14.1	EF-hand calcium binding domain 2
9045	0.55	RPL14	ribosomal protein L14
7536	0.51	ZNF162	splicing factor 1
84833	0.50	USMG5	upregulated during skeletal muscle growth 5 homolog (mouse)
143570	0.49	XRRA1	X-ray radiation resistance associated 1
155368	0.46	WBSCR27	Williams Beuren syndrome chromosome region 27
282991	0.46	RP11-316M21.4	biogenesis of lysosome-related organelles complex-1, subunit 2
7311	0.43	UBA52	ubiquitin A-52 residue ribosomal protein fusion product 1
133383	0.42	MGC33648	hypothetical protein MGC33648
829	0.41	CAZ1	capping protein (actin filament) muscle Z-line, alpha 1
84545	0.41	MRPL43	mitochondrial ribosomal protein L43
84641	0.41	MGC117350	hippocampus abundant transcript-like 1
3105	0.38	HLA-A	major histocompatibility complex, class I, A
54879	0.38	STLR	suppression of tumorigenicity 7 like
858	0.38	MGC12294	caveolin 2
10494	0.37	YSK1	serine/threonine kinase 25 (STE20 homolog, yeast)
1965	0.37	EIF2S1	eukaryotic translation initiation factor 2, subunit 1 alpha, 35kDa
4725	0.37	NDUFS5	NADH dehydrogenase (ubiquinone) Fe-S protein 5, 15kDa (NADH-coenzyme Q reductase)
55556	0.37	TYMSAS	enolase superfamily member 1
3127	0.36	HLA-DRB5	major histocompatibility complex, class II, DR beta 5
5190	0.36	PXAAA1	peroxisomal biogenesis factor 6
162394	0.35	SLFN5	schlafen family member 5
219927	0.35	MRPL21	mitochondrial ribosomal protein L21
439921	0.35	TMAP1	matrix-remodelling associated 7
6199	0.35	STK14B	ribosomal protein S6 kinase, 70kDa, polypeptide 2
84680	0.35	PHACS	1-aminocyclopropane-1-carboxylate synthase
10233	0.31	LRRC23	leucine rich repeat containing 23
55755	0.31	MCPH3	CDK5 regulatory subunit associated protein 2
348094	0.30	MGC120307	ankyrin repeat and death domain containing 1A
9419	0.30	HSPC139	postsynaptic protein CRIPT
51101	0.29	CGI-62	chromosome 8 open reading frame 70
84314	0.29	TMEM107	transmembrane protein 107
51074	0.28	MMRP19	APAF1 interacting protein
64847	0.28	Tisp78	spermatogenesis associated 20
115024	0.27	NT5C3L	5'-nucleotidase, cytosolic III-like
255403	0.26	ZNF718	zinc finger protein 718
261726	0.26	TIPRL	TIP41, TOR signalling pathway regulator-like (S. cerevisiae)
2635	0.26	GBP3	guanylate binding protein 3
349565	0.26	PNAT3	nicotinamide nucleotide adenylyltransferase 3
389362	0.26	RP11-506K6.3	hypothetical LOC389362
87769	0.26	RP11-151A6.2	hypothetical protein BC004360
93100	0.26	PP3856	nicotinate phosphoribosyltransferase domain containing 1
23593	0.25	SOUL	heme binding protein 2
26519	0.25	TIMM10	translocase of inner mitochondrial membrane 10 homolog (yeast)
10438	0.24	SUNCOR	nuclear DNA-binding protein
318	0.24	NUDT2	nudix (nucleoside diphosphate linked moiety X)-type motif 2
3652	0.24	IPP	intracisternal A particle-promoted polypeptide
55871	0.24	CBWD1	COBW domain containing 1
81889	0.24	YISKL	fumarylacetoacetate hydrolase domain containing 1
11118	0.23	BTN3A2	butyrophilin, subfamily 3, member A2
131076	0.23	FLJ33273	coiled-coil domain containing 58
25895	0.23	FAM119B	family with sequence similarity 119, member B
55228	0.23	FLJ10781	hypothetical protein FLJ10781
84930	0.23	THC2	microtubule associated serine/threonine kinase-like
27068	0.22	SID6-306	pyrophosphatase (inorganic) 2
64858	0.22	SNM1B	DNA cross-link repair 1B (PSO2 homolog, S. cerevisiae)
152815	0.21	THAP6	THAP domain containing 6

3117	0.21	HLA-DQA	major histocompatibility complex, class II, DQ alpha 1
400566	0.21	LOC400566	hypothetical gene supported by AK128660
51144	0.21	KAR	hydroxysteroid (17-beta) dehydrogenase 12
54014	0.21	WDR9	bromodomain and WD repeat domain containing 1
6284	0.21	S100A13	S100 calcium binding protein A13
90273	0.21	R29124_1	carcinoembryonic antigen-related cell adhesion molecule 21
10781	0.20	ZNF266	zinc finger protein 266
126272	0.20	FLJ38944	EID-2-like inhibitor of differentiation-3
25885	0.20	RPO1-4	polymerase (RNA) I polypeptide A, 194kDa
2944	0.20	MU	glutathione S-transferase M1
4552	0.20	MTRR	5-methyltetrahydrofolate-homocysteine methyltransferase reductase
51097	0.20	SCCPDH	saccharopine dehydrogenase (putative)
5434	0.20	XAP4	polymerase (RNA) II (DNA directed) polypeptide E, 25kDa
6217	0.20	RPS16	ribosomal protein S16
8904	0.20	MGC1142	copine I
10455	0.19	PECI	peroxisomal D3,D2-enoyl-CoA isomerase
145173	0.19	Gal-T	beta 1,3-galactosyltransferase-like
1512	0.19	minichain	cathepsin H
3700	0.19	PK120	inter-alpha (globulin) inhibitor H4 (plasma Kallikrein-sensitive glycoprotein)
6168	0.19	RPL37A	ribosomal protein L37a
7390	0.19	UROS	uroporphyrinogen III synthase (congenital erythropoietic porphyria)
92106	0.19	OXNAD1	oxidoreductase NAD-binding domain containing 1
146279	0.18	FLJ32871	hypothetical protein FLJ32871
151963	0.18	C3orf59	chromosome 3 open reading frame 59
22927	0.18	SERBP1L	hyaluronan binding protein 4
283232	0.18	TMEM80	transmembrane protein 80
4605	0.18	MYBL2	v-myb myeloblastosis viral oncogene homolog (avian)-like 2
51657	0.18	STYXL1	serine/threonine/tyrosine interacting-like 1
57602	0.18	USP36	ubiquitin specific peptidase 36
63897	0.18	FLJ22087	amplified in breast cancer 1
79741	0.18	FLJ13031	chromosome 10 open reading frame 68
84328	0.18	MGC15436	leucine zipper and CTNNBIP1 domain containing
84617	0.18	TUBB6	tubulin, beta 6
953	0.18	NTPDase-1	ectonucleoside triphosphate diphosphohydrolase 1
127255	0.17	MGC22773	leucine rich repeat containing 44
