# Supplementary material for: Robust Prediction of Expression Differences among Human Individuals Using Only Genotype Information
Source: PLoS Genet. 2013 Mar 28;9(3):e1003396. doi: 10.1371/journal.pgen.1003396 (PMC3610805; doi:10.1371/journal.pgen.1003396)
Supplement: Table S12 — The list of the 100 top-predicted genes of the Elastic-Net model in the Intra-Pop cross-validation scheme. (PDF) [file pgen.1003396.s016.pdf]

Table S12. The list of the 100 top-predicted genes of the Elastic-Net model in the Intra-Pop cross-validation scheme

Entrez ID	Mean Test R^2	Gene Symbol	Gene Description
91612	0.82	My015	churchill domain containing 1
94101	0.80	ORMDL1	ORM1-like 1 (S. cerevisiae)
3123	0.79	HLA-DRB1	major histocompatibility complex, class II, DR beta 1
93035	0.76	PKHDL1	polycystic kidney and hepatic disease 1 (autosomal recessive)-like 1
6231	0.75	RPS26	ribosomal protein S26
9045	0.74	RPL14	ribosomal protein L14
400566	0.73	LOC400566	hypothetical gene supported by AK128660
5434	0.71	XAP4	polymerase (RNA) II (DNA directed) polypeptide E, 25kDa
158160	0.70	MGC88684	hydroxysteroid (17-beta) dehydrogenase 7 pseudogene 2
3105	0.69	HLA-A	major histocompatibility complex, class I, A
9317	0.67	RPR-1	phosphotriesterase related
7311	0.64	UBA52	ubiquitin A-52 residue ribosomal protein fusion product 1
439921	0.63	TMAP1	matrix-remodelling associated 7
7536	0.62	ZNF162	splicing factor 1
84833	0.62	USMG5	upregulated during skeletal muscle growth 5 homolog (mouse)
3663	0.61	IRF5	interferon regulatory factor 5
27004	0.59	TNG2	T-cell leukemia/lymphoma 6
55228	0.58	FLJ10781	hypothetical protein FLJ10781
3127	0.56	HLA-DRB5	major histocompatibility complex, class II, DR beta 5
64847	0.56	Tisp78	spermatogenesis associated 20
3117	0.55	HLA-DQA	major histocompatibility complex, class II, DQ alpha 1
84288	0.55	RP11-290P14.1	EF-hand calcium binding domain 2
10494	0.54	YSK1	serine/threonine kinase 25 (STE20 homolog, yeast)
84680	0.53	PHACS	1-aminocyclopropane-1-carboxylate synthase
143570	0.52	XRRA1	X-ray radiation resistance associated 1
54879	0.51	STLR	suppression of tumorigenicity 7 like
93100	0.50	PP3856	nicotinate phosphoribosyltransferase domain containing 1
25902	0.49	MTHFD1L	methylenetetrahydrofolate dehydrogenase (NADP+ dependent) 1-like
829	0.49	CAZ1	capping protein (actin filament) muscle Z-line, alpha 1
131076	0.48	FLJ33273	coiled-coil domain containing 58
2944	0.48	MU	glutathione S-transferase M1
9623	0.47	TML1	T-cell leukemia/lymphoma 1B
90273	0.47	R29124_1	carcinoembryonic antigen-related cell adhesion molecule 21
10438	0.47	SUNCOR	nuclear DNA-binding protein
133383	0.46	MGC33648	hypothetical protein MGC33648
348094	0.46	MGC120307	ankyrin repeat and death domain containing 1A
79618	0.45	PBHNF	homeobox containing 1
155368	0.45	WBSCR27	Williams Beuren syndrome chromosome region 27
87769	0.45	RP11-151A6.2	hypothetical protein BC004360
5190	0.45	PXAAA1	peroxisomal biogenesis factor 6
7367	0.44	UGT2B17	UDP glucuronosyltransferase 2 family, polypeptide B17
55556	0.44	TYMSAS	enolase superfamily member 1
162394	0.44	SLFN5	schlafen family member 5
127255	0.43	MGC22773	leucine rich repeat containing 44
349565	0.43	PNAT3	nicotinamide nucleotide adenylyltransferase 3
84545	0.43	MRPL43	mitochondrial ribosomal protein L43
1965	0.43	EIF2S1	eukaryotic translation initiation factor 2, subunit 1 alpha, 35kDa
6199	0.43	STK14B	ribosomal protein S6 kinase, 70kDa, polypeptide 2
55871	0.41	CBWD1	COBW domain containing 1
84641	0.41	MGC117350	hippocampus abundant transcript-like 1
219927	0.41	MRPL21	mitochondrial ribosomal protein L21
6168	0.40	RPL37A	ribosomal protein L37a
261726	0.40	TIPRL	TIP41, TOR signalling pathway regulator-like (S. cerevisiae)
10077	0.39	TSSC6	tetraspanin 32
4725	0.39	NDUFS5	NADH dehydrogenase (ubiquinone) Fe-S protein 5, 15kDa (NADH-coenzyme Q reductase)
152815	0.39	THAP6	THAP domain containing 6
55755	0.39	MCPH3	CDK5 regulatory subunit associated protein 2
26519	0.38	TIMM10	translocase of inner mitochondrial membrane 10 homolog (yeast)
51074	0.38	MMRP19	APAF1 interacting protein
9419	0.38	HSPC139	postsynaptic protein CRIPT
2635	0.38	GBP3	guanylate binding protein 3
282991	0.37	RP11-316M21.4	biogenesis of lysosome-related organelles complex-1, subunit 2
151963	0.37	C3orf59	chromosome 3 open reading frame 59
118980	0.36	SFXN2	sideroflexin 2

10352	0.36	WARS2	tryptophanyl tRNA synthetase 2 (mitochondrial)
10233	0.35	LRRC23	leucine rich repeat containing 23
113386	0.34	LOC113386	similar to envelope protein
80817	0.34	PS1TP3	KIAA1712
64858	0.33	SNM1B	DNA cross-link repair 1B (PSO2 homolog, S. cerevisiae)
8034	0.33	SLC25A16	solute carrier family 25 (mitochondrial carrier; Graves disease autoantigen), member 16
4552	0.33	MTRR	5-methyltetrahydrofolate-homocysteine methyltransferase reductase
1838	0.33	MGC57126	dystrobrevin, beta
255403	0.33	ZNF718	zinc finger protein 718
8904	0.33	MGC1142	copine I
8312	0.33	MGC52315	axin 1
115024	0.32	NT5C3L	5'-nucleotidase, cytosolic III-like
1635	0.32	MGC111062	dCMP deaminase
23008	0.32	KIAA0265	KIAA0265 protein
27068	0.32	SID6-306	pyrophosphatase (inorganic) 2
80162	0.32	MGC129859	ATH1, acid trehalase-like 1 (yeast)
389362	0.32	RP11-506K6.3	hypothetical LOC389362
196446	0.32	MGC126869	chromosome 12 open reading frame 28
84328	0.32	MGC15436	leucine zipper and CTNNBIP1 domain containing
29928	0.31	TIMM22	translocase of inner mitochondrial membrane 22 homolog (yeast)
7773	0.31	ZNF230	zinc finger protein 230
80221	0.30	FLJ20920	hypothetical protein FLJ20920
10953	0.30	TOMM34	translocase of outer mitochondrial membrane 34
116028	0.30	MGC24665	hypothetical protein MGC24665
25895	0.30	FAM119B	family with sequence similarity 119, member B
80008	0.30	TMEM156	transmembrane protein 156
5411	0.30	SDK3	pinin, desmosome associated protein
80315	0.30	KIAA1673	cytoplasmic polyadenylation element binding protein 4
51097	0.30	SCCPDH	saccharopine dehydrogenase (putative)
116540	0.30	MRPL53	mitochondrial ribosomal protein L53
3652	0.30	IPP	intracisternal A particle-promoted polypeptide
79762	0.30	RP11-322F10.4	chromosome 1 open reading frame 115
25885	0.29	RPO1-4	polymerase (RNA) I polypeptide A, 194kDa
54014	0.29	WDR9	bromodomain and WD repeat domain containing 1
6217	0.29	RPS16	ribosomal protein S16
11118	0.29	BTN3A2	butyrophilin, subfamily 3, member A2
