# Supplementary material for: Robust Prediction of Expression Differences among Human Individuals Using Only Genotype Information
Source: PLoS Genet. 2013 Mar 28;9(3):e1003396. doi: 10.1371/journal.pgen.1003396 (PMC3610805; doi:10.1371/journal.pgen.1003396)
Supplement: Table S13 — The list of the 100 top-predicted genes of the Single-SNP model in the Intra-Pop cross-validation scheme. (PDF) [file pgen.1003396.s017.pdf]

Table S13. The list of the 100 top-predicted genes of the Single-SNP model in the Intra-Pop cross-validation scheme

Entrez ID	Mean Test R^2	Gene Symbol	Gene Description
7311	0.84	UBA52	ubiquitin A-52 residue ribosomal protein fusion product 1
91612	0.82	My015	churchill domain containing 1
6231	0.77	RPS26	ribosomal protein S26
9045	0.77	RPL14	ribosomal protein L14
27004	0.75	TNG2	T-cell leukemia/lymphoma 6
84833	0.75	USMG5	upregulated during skeletal muscle growth 5 homolog (mouse)
3117	0.74	HLA-DQA	major histocompatibility complex, class II, DQ alpha 1
94101	0.73	ORMDL1	ORM1-like 1 (S. cerevisiae)
5434	0.70	XAP4	polymerase (RNA) II (DNA directed) polypeptide E, 25kDa
84680	0.70	PHACS	1-aminocyclopropane-1-carboxylate synthase
84641	0.69	MGC117350	hippocampus abundant transcript-like 1
9317	0.69	RPR-1	phosphotriesterase related
282991	0.68	RP11-316M21.4	biogenesis of lysosome-related organelles complex-1, subunit 2
143570	0.65	XRRA1	X-ray radiation resistance associated 1
158160	0.65	MGC88684	hydroxysteroid (17-beta) dehydrogenase 7 pseudogene 2
64847	0.64	Tisp78	spermatogenesis associated 20
79618	0.64	PBHNF	homeobox containing 1
93035	0.64	PKHDL1	polycystic kidney and hepatic disease 1 (autosomal recessive)-like 1
3663	0.63	IRF5	interferon regulatory factor 5
10494	0.62	YSK1	serine/threonine kinase 25 (STE20 homolog, yeast)
83543	0.62	MGC29466	chromosome 9 open reading frame 58
348094	0.60	MGC120307	ankyrin repeat and death domain containing 1A
858	0.59	MGC12294	caveolin 2
25902	0.59	MTHFD1L	methylenetetrahydrofolate dehydrogenase (NADP+ dependent) 1-like
6168	0.58	RPL37A	ribosomal protein L37a
55755	0.55	MCPH3	CDK5 regulatory subunit associated protein 2
5190	0.55	PXAAA1	peroxisomal biogenesis factor 6
4731	0.54	NDUFV3	NADH dehydrogenase (ubiquinone) flavoprotein 3, 10kDa
3127	0.54	HLA-DRB5	major histocompatibility complex, class II, DR beta 5
93100	0.54	PP3856	nicotinate phosphoribosyltransferase domain containing 1
90273	0.53	R29124_1	carcinoembryonic antigen-related cell adhesion molecule 21
9623	0.53	TML1	T-cell leukemia/lymphoma 1B
155368	0.52	WBSCR27	Williams Beuren syndrome chromosome region 27
439921	0.51	TMAP1	matrix-remodelling associated 7
23593	0.50	SOUL	heme binding protein 2
349565	0.50	PNAT3	nicotinamide nucleotide adenylyltransferase 3
84288	0.49	RP11-290P14.1	EF-hand calcium binding domain 2
829	0.49	CAZ1	capping protein (actin filament) muscle Z-line, alpha 1
1965	0.48	EIF2S1	eukaryotic translation initiation factor 2, subunit 1 alpha, 35kDa
87769	0.48	RP11-151A6.2	hypothetical protein BC004360
7536	0.48	ZNF162	splicing factor 1
133383	0.47	MGC33648	hypothetical protein MGC33648
3105	0.47	HLA-A	major histocompatibility complex, class I, A
54879	0.47	STLR	suppression of tumorigenicity 7 like
3118	0.47	HLA-DXA	major histocompatibility complex, class II, DQ alpha 2
219927	0.47	MRPL21	mitochondrial ribosomal protein L21
400566	0.47	LOC400566	hypothetical gene supported by AK128660
131076	0.47	FLJ33273	coiled-coil domain containing 58
6199	0.46	STK14B	ribosomal protein S6 kinase, 70kDa, polypeptide 2
162394	0.46	SLFN5	schlafen family member 5
196446	0.46	MGC126869	chromosome 12 open reading frame 28
84545	0.45	MRPL43	mitochondrial ribosomal protein L43
255403	0.44	ZNF718	zinc finger protein 718
10438	0.44	SUNCOR	nuclear DNA-binding protein
197322	0.44	LOC197322	hypothetical protein LOC197322
10905	0.44	MAN1B	mannosidase, alpha, class 1A, member 2
11147	0.43	HHLA3	HERV-H LTR-associating 3
27068	0.43	SID6-306	pyrophosphatase (inorganic) 2
283726	0.43	DKFZp666G057	hypothetical protein DKFZp666G057
84221	0.43	MGC99490	chromosome 21 open reading frame 56
80221	0.42	FLJ20920	hypothetical protein FLJ20920
80315	0.42	KIAA1673	cytoplasmic polyadenylation element binding protein 4
127255	0.42	MGC22773	leucine rich repeat containing 44
91687	0.42	RP3-383J4.1	centromere protein L
8312	0.42	MGC52315	axin 1

8904	0.42	MGC1142	copine I
55228	0.40	FLJ10781	hypothetical protein FLJ10781
55871	0.40	CBWD1	COBW domain containing 1
118980	0.40	SFXN2	sideroflexin 2
7773	0.40	ZNF230	zinc finger protein 230
51101	0.40	CGI-62	chromosome 8 open reading frame 70
3123	0.40	HLA-DRB1	major histocompatibility complex, class II, DR beta 1
26519	0.39	TIMM10	translocase of inner mitochondrial membrane 10 homolog (yeast)
25895	0.39	FAM119B	family with sequence similarity 119, member B
11118	0.38	BTN3A2	butyrophilin, subfamily 3, member A2
115024	0.38	NT5C3L	5'-nucleotidase, cytosolic III-like
25885	0.37	RPO1-4	polymerase (RNA) I polypeptide A, 194kDa
7639	0.37	ZNF85	zinc finger protein 85
8878	0.37	ZIP3	sequestosome 1
9882	0.37	TBC1D4	TBC1 domain family, member 4
116028	0.37	MGC24665	hypothetical protein MGC24665
4725	0.36	NDUFS5	NADH dehydrogenase (ubiquinone) Fe-S protein 5, 15kDa (NADH-coenzyme Q reductase)
1635	0.36	MGC111062	dCMP deaminase
80008	0.36	TMEM156	transmembrane protein 156
10233	0.36	LRRC23	leucine rich repeat containing 23
5411	0.36	SDK3	pinin, desmosome associated protein
318	0.35	NUDT2	nudix (nucleoside diphosphate linked moiety X)-type motif 2
9501	0.34	RPH3AL	rabphilin 3A-like (without C2 domains)
151246	0.34	TRIPIN	shugoshin-like 2 (S. pombe)
261726	0.34	TIPRL	TIP41, TOR signalling pathway regulator-like (S. cerevisiae)
51122	0.34	MGC57611	COMM domain containing 2
10953	0.34	TOMM34	translocase of outer mitochondrial membrane 34
6217	0.34	RPS16	ribosomal protein S16
55556	0.34	TYMSAS	enolase superfamily member 1
10412	0.34	YR-29	TGF beta-inducible nuclear protein 1
63897	0.34	FLJ22087	amplified in breast cancer 1
7367	0.34	UGT2B17	UDP glucuronosyltransferase 2 family, polypeptide B17
55278	0.34	QRSL1	glutaminyl-tRNA synthase (glutamine-hydrolyzing)-like 1
81544	0.34	PP1665	glycerophosphodiester phosphodiesterase domain containing 5
132001	0.33	MGC16471	chromosome 3 open reading frame 31
