# Supplementary material for: Robust Prediction of Expression Differences among Human Individuals Using Only Genotype Information
Source: PLoS Genet. 2013 Mar 28;9(3):e1003396. doi: 10.1371/journal.pgen.1003396 (PMC3610805; doi:10.1371/journal.pgen.1003396)
Supplement: Table S14 — For each cross-validation scheme and model, shown is the percentage of genes (with test R∧2> = 0.05) where the model had the best predictive power. (PDF) [file pgen.1003396.s018.pdf]

Table S14. for each cross-validation scheme and model, shown is the percentage of genes (with test R^2 >= 0.05) where the model had the best predictive power

	KNN	Elastic-Net	Combined	Single-SNP
Cross-pop	9%	39%	16%	34%
Mixed-pop	3%	37%	18%	41%
Intra-pop	3%	47%	22%	26%
