# Supplementary material for: Robust Prediction of Expression Differences among Human Individuals Using Only Genotype Information
Source: PLoS Genet. 2013 Mar 28;9(3):e1003396. doi: 10.1371/journal.pgen.1003396 (PMC3610805; doi:10.1371/journal.pgen.1003396)
Supplement: Table S15 — Full list of all genomic features complied for this paper. (PDF) [file pgen.1003396.s019.pdf]

Table S15. Full list of all genomic features complied for this paper

#	Genomic Feature	Type	Description / source
1	Promoter	Binary	SNP Location in gene
2	5UTR	Binary	SNP Location in gene
3	CDS	Binary	SNP Location in gene
4	Intron	Binary	SNP Location in gene
5	3UTR	Binary	SNP Location in gene
6	GC-50-Low	Binary	GC% < 0.3 in the flanking 50bp of the SNP
7	GC-50-MedLow	Binary	0.3 < GC% < 0.5 in the flanking 50bp of the SNP
8	GC-50-MedHigh	Binary	0.5 < GC% < 0.7 in the flanking 50bp of the SNP
9	GC-50-High	Binary	GC% > 0.7 in the flanking 50bp of the SNP
10	GC-100-Low	Binary	GC% < 0.3 in the flanking 100bp of the SNP
11	GC-100-MedLow	Binary	0.3 < GC% < 0.5 in the flanking 100bp of the SNP
12	GC-100-MedHigh	Binary	0.5 < GC% < 0.7 in the flanking 100bp of the SNP
13	GC-100-High	Binary	GC% > 0.7 in the flanking 100bp of the SNP
14	Histone-H3K4me1	Cont.	Different Histone markers, taken from http://dir.nhlbi.nih.gov/papers/lmi/epigenomes/hgtcell.aspx
15	Histone-H3K4me2	Cont.	Different Histone markers, taken from http://dir.nhlbi.nih.gov/papers/lmi/epigenomes/hgtcell.aspx
16	Histone-H3K4me3	Cont.	Different Histone markers, taken from http://dir.nhlbi.nih.gov/papers/lmi/epigenomes/hgtcell.aspx
17	Histone-H3K9me1	Cont.	Different Histone markers, taken from http://dir.nhlbi.nih.gov/papers/lmi/epigenomes/hgtcell.aspx
18	Histone-H3K9me2	Cont.	Different Histone markers, taken from http://dir.nhlbi.nih.gov/papers/lmi/epigenomes/hgtcell.aspx
19	Histone-H3K9me3	Cont.	Different Histone markers, taken from http://dir.nhlbi.nih.gov/papers/lmi/epigenomes/hgtcell.aspx
20	Histone-H3K27me1	Cont.	Different Histone markers, taken from http://dir.nhlbi.nih.gov/papers/lmi/epigenomes/hgtcell.aspx
21	Histone-H3K27me2	Cont.	Different Histone markers, taken from http://dir.nhlbi.nih.gov/papers/lmi/epigenomes/hgtcell.aspx
22	Histone-H3K27me3	Cont.	Different Histone markers, taken from http://dir.nhlbi.nih.gov/papers/lmi/epigenomes/hgtcell.aspx
23	Histone-H3K36me1	Cont.	Different Histone markers, taken from http://dir.nhlbi.nih.gov/papers/lmi/epigenomes/hgtcell.aspx
24	Histone-H3K36me3	Cont.	Different Histone markers, taken from http://dir.nhlbi.nih.gov/papers/lmi/epigenomes/hgtcell.aspx
25	Histone-H3K79me1	Cont.	Different Histone markers, taken from http://dir.nhlbi.nih.gov/papers/lmi/epigenomes/hgtcell.aspx
26	Histone-H3K79me2	Cont.	Different Histone markers, taken from http://dir.nhlbi.nih.gov/papers/lmi/epigenomes/hgtcell.aspx

27	Histone-H3K79me3	Cont.	Different Histone markers, taken from http://dir.nhlbi.nih.gov/papers/lmi/epigenomes/hgtcell.aspx
28	Histone-H3R2me1	Cont.	Different Histone markers, taken from http://dir.nhlbi.nih.gov/papers/lmi/epigenomes/hgtcell.aspx
29	Histone-H3R2me2	Cont.	Different Histone markers, taken from http://dir.nhlbi.nih.gov/papers/lmi/epigenomes/hgtcell.aspx
30	Histone-H4K20me1	Cont.	Different Histone markers, taken from http://dir.nhlbi.nih.gov/papers/lmi/epigenomes/hgtcell.aspx
31	Histone-H4K20me3	Cont.	Different Histone markers, taken from http://dir.nhlbi.nih.gov/papers/lmi/epigenomes/hgtcell.aspx
32	Histone-H4R3me2	Cont.	Different Histone markers, taken from http://dir.nhlbi.nih.gov/papers/lmi/epigenomes/hgtcell.aspx
33	Histone-H2BK5me1	Cont.	Different Histone markers, taken from http://dir.nhlbi.nih.gov/papers/lmi/epigenomes/hgtcell.aspx
34	Histone-H2AZ	Cont.	Different Histone markers, taken from http://dir.nhlbi.nih.gov/papers/lmi/epigenomes/hgtcell.aspx
35	PolII	Cont.	Different Histone markers, taken from http://dir.nhlbi.nih.gov/papers/lmi/epigenomes/hgtcell.aspx
36	CTCF	Cont.	Different Histone markers, taken from http://dir.nhlbi.nih.gov/papers/lmi/epigenomes/hgtcell.aspx
37	HistoneAcetyl-H2AK5ac	Cont.	Different Histone markers, taken from http://dir.nhlbi.nih.gov/papers/lmi/epigenomes/hgtcell.aspx
38	HistoneAcetyl-H2AK9ac	Cont.	Different Histone markers, taken from http://dir.nhlbi.nih.gov/papers/lmi/epigenomes/hgtcell.aspx
39	HistoneAcetyl-H2BK120ac	Cont.	Different Histone markers, taken from http://dir.nhlbi.nih.gov/papers/lmi/epigenomes/hgtcell.aspx
40	HistoneAcetyl-H2BK12ac	Cont.	Different Histone markers, taken from http://dir.nhlbi.nih.gov/papers/lmi/epigenomes/hgtcell.aspx
41	HistoneAcetyl-H2BK20ac	Cont.	Different Histone markers, taken from http://dir.nhlbi.nih.gov/papers/lmi/epigenomes/hgtcell.aspx
42	HistoneAcetyl-H2BK5ac	Cont.	Different Histone markers, taken from http://dir.nhlbi.nih.gov/papers/lmi/epigenomes/hgtcell.aspx
43	HistoneAcetyl-H3K14ac	Cont.	Different Histone markers, taken from http://dir.nhlbi.nih.gov/papers/lmi/epigenomes/hgtcell.aspx
44	HistoneAcetyl-H3K18ac	Cont.	Different Histone markers, taken from http://dir.nhlbi.nih.gov/papers/lmi/epigenomes/hgtcell.aspx
45	HistoneAcetyl-H3K23ac	Cont.	Different Histone markers, taken from http://dir.nhlbi.nih.gov/papers/lmi/epigenomes/hgtcell.aspx

46	HistoneAcetyl-H3K27ac	Cont.	Different Histone markers, taken from http://dir.nhlbi.nih.gov/papers/lmi/epigenomes/hgtcell.aspx
47	HistoneAcetyl-H3K36ac	Cont.	Different Histone markers, taken from http://dir.nhlbi.nih.gov/papers/lmi/epigenomes/hgtcell.aspx
48	HistoneAcetyl-H3K4ac	Cont.	Different Histone markers, taken from http://dir.nhlbi.nih.gov/papers/lmi/epigenomes/hgtcell.aspx
49	HistoneAcetyl-H3K9ac	Cont.	Different Histone markers, taken from http://dir.nhlbi.nih.gov/papers/lmi/epigenomes/hgtcell.aspx
50	HistoneAcetyl-H4K12ac	Cont.	Different Histone markers, taken from http://dir.nhlbi.nih.gov/papers/lmi/epigenomes/hgtcell.aspx
51	HistoneAcetyl-H4K16ac	Cont.	Different Histone markers, taken from http://dir.nhlbi.nih.gov/papers/lmi/epigenomes/hgtcell.aspx
52	HistoneAcetyl-H4K5ac	Cont.	Different Histone markers, taken from http://dir.nhlbi.nih.gov/papers/lmi/epigenomes/hgtcell.aspx
53	HistoneAcetyl-H4K8ac	Cont.	Different Histone markers, taken from http://dir.nhlbi.nih.gov/papers/lmi/epigenomes/hgtcell.aspx
54	HistoneAcetyl-H4K91ac	Cont.	Different Histone markers, taken from http://dir.nhlbi.nih.gov/papers/lmi/epigenomes/hgtcell.aspx
55	1_Active_Promoter	Binary	Encode track from the paper by Ernst and Kellis: doi:10.1038/nbt.1662
56	2_Weak_Promoter	Binary	Encode track from the paper by Ernst and Kellis: doi:10.1038/nbt.1662
57	3_Poised_Promoter	Binary	Encode track from the paper by Ernst and Kellis: doi:10.1038/nbt.1662
58	4_Strong_Enhancer	Binary	Encode track from the paper by Ernst and Kellis: doi:10.1038/nbt.1662
59	5_Strong_Enhancer	Binary	Encode track from the paper by Ernst and Kellis: doi:10.1038/nbt.1662
60	6_Weak_Enhancer	Binary	Encode track from the paper by Ernst and Kellis: doi:10.1038/nbt.1662
61	7_Weak_Enhancer	Binary	Encode track from the paper by Ernst and Kellis: doi:10.1038/nbt.1662
62	8_Insulator	Binary	Encode track from the paper by Ernst and Kellis: doi:10.1038/nbt.1662
63	9_Txn_Transition	Binary	Encode track from the paper by Ernst and Kellis: doi:10.1038/nbt.1662
64	10_Txn_Elongation	Binary	Encode track from the paper by Ernst and Kellis: doi:10.1038/nbt.1662
65	11_Weak_Txn	Binary	Encode track from the paper by Ernst and Kellis: doi:10.1038/nbt.1662
66	12_Repressed	Binary	Encode track from the paper by Ernst and Kellis: doi:10.1038/nbt.1662
67	13_Heterochrom_lo	Binary	Encode track from the paper by Ernst and Kellis: doi:10.1038/nbt.1662
68	14_Repetitive_CNV	Binary	Encode track from the paper by Ernst and Kellis: doi:10.1038/nbt.1662

69	15_Repetitive_CNV	Binary	Encode track from the paper by Ernst and Kellis: doi:10.1038/nbt.1662
70	CONS-1-Low	Binary	UCSC conservation track, conservation < 0.1
71	CONS-1-MedLow	Binary	UCSC conservation track, 0.1 < conservation < 0.3
72	CONS-1-MedHigh	Binary	UCSC conservation track, 0.3 < conservation < 0.6
73	CONS-1-High	Binary	UCSC conservation track, conservation > 0.6
74	CONS-10-Low	Binary	UCSC conservation track, average conservation in 10 flanking bp < 0.1
75	CONS-10-MedLow	Binary	UCSC conservation track, 0.1 < average conservation in 10 flanking bp < 0.3
76	CONS-10-MedHigh	Binary	UCSC conservation track, 0.3 < average conservation in 10 flanking bp < 0.6
77	CONS-10-High	Binary	UCSC conservation track, average conservation in 10 flanking bp > 0.6
78	MIRNA_TS	Binary	SNP located in microRNA target site by TargetScan prediction (http://www.targetscan.org/)
79	MIRNA_PITA	Binary	SNP located in microRNA target site by PITA prediction (http://genie.weizmann.ac.il/pubs/mir07/mir07_prediction.html)
80	CPG	Binary	UCSC CpG Island track
81	CHROMATIN_AVG	Cont.	Average nucleosome occupancy predicted using model (http://genie.weizmann.ac.il/software/nucleo_prediction.html)
82	REG_POTEN	Cont.	UCSC regulatory potential track
83	ENCODE_TFBS_CLUSTERED	Binary	Encode track for Clustered transcription factor binding sites
84	TFBS_CENT	Binary	transcription factor binding sites from http://centipede.uchicago.edu/
85	MethylSeq_Gm12878	Binary	Methylation status, taken from ENCODE, Hudson Alpha GM12878/91/92
86	MethylSeq_Gm12891	Binary	Methylation status, taken from ENCODE, Hudson Alpha GM12878/91/92
87	MethylSeq_Gm12892	Binary	Methylation status, taken from ENCODE, Hudson Alpha GM12878/91/92
88	ChipSeq_Ctcf	Cont.	Broad ChipSeq, taken from ENCODE
89	ChipSeq_H3k27ac	Cont.	Broad ChipSeq, taken from ENCODE
90	ChipSeq_H3k27me3	Cont.	Broad ChipSeq, taken from ENCODE
91	ChipSeq_H3k36me3	Cont.	Broad ChipSeq, taken from ENCODE
92	ChipSeq_H3k4me1	Cont.	Broad ChipSeq, taken from ENCODE
93	ChipSeq_H3k4me2	Cont.	Broad ChipSeq, taken from ENCODE
94	ChipSeq_H3k4me3	Cont.	Broad ChipSeq, taken from ENCODE
95	ChipSeq_H3k9ac	Cont.	Broad ChipSeq, taken from ENCODE
96	ChipSeq_H4k20me1	Cont.	Broad ChipSeq, taken from ENCODE
97	TSS_UP_0_1000	Binary	TSS/TES distance bin, gene coordinates taken from UCSC
98	TES_UP_0_1000	Binary	TSS/TES distance bin, gene coordinates taken from UCSC
99	TSS_DOWN_0_1000	Binary	TSS/TES distance bin, gene coordinates taken from UCSC

100	TES_DOWN_0_1000	Binary	TSS/TES distance bin, gene coordinates taken from UCSC
101	TSS_UP_1000_5000	Binary	TSS/TES distance bin, gene coordinates taken from UCSC
102	TES_UP_1000_5000	Binary	TSS/TES distance bin, gene coordinates taken from UCSC
103	TSS_DOWN_1000_5000	Binary	TSS/TES distance bin, gene coordinates taken from UCSC
104	TES_DOWN_1000_5000	Binary	TSS/TES distance bin, gene coordinates taken from UCSC
105	TSS_UP_5000_15000	Binary	TSS/TES distance bin, gene coordinates taken from UCSC
106	TES_UP_5000_15000	Binary	TSS/TES distance bin, gene coordinates taken from UCSC
107	TSS_DOWN 5000_15000	Binary	TSS/TES distance bin, gene coordinates taken from UCSC
108	TES_DOWN 5000_15000	Binary	TSS/TES distance bin, gene coordinates taken from UCSC
109	TSS_UP_15000_50000	Binary	TSS/TES distance bin, gene coordinates taken from UCSC
110	TES_UP_15000_50000	Binary	TSS/TES distance bin, gene coordinates taken from UCSC
111	TSS_DOWN 15000_50000	Binary	TSS/TES distance bin, gene coordinates taken from UCSC
112	TES_DOWN 15000_50000	Binary	TSS/TES distance bin, gene coordinates taken from UCSC
113	TSS_UP_50000_100000	Binary	TSS/TES distance bin, gene coordinates taken from UCSC
114	TES_UP_50000_100000	Binary	TSS/TES distance bin, gene coordinates taken from UCSC
115	TSS_DOWN 50000_100000	Binary	TSS/TES distance bin, gene coordinates taken from UCSC
116	TES_DOWN 50000_100000	Binary	TSS/TES distance bin, gene coordinates taken from UCSC
117	Entropy	Cont.	SNP entropy across all individuals (see methods)
