# Supplementary material for: Robust Prediction of Expression Differences among Human Individuals Using Only Genotype Information
Source: PLoS Genet. 2013 Mar 28;9(3):e1003396. doi: 10.1371/journal.pgen.1003396 (PMC3610805; doi:10.1371/journal.pgen.1003396)
Supplement: Table S16 — Enriched and depleted genomic features in multi-SNP models. Shown are SNP genomic features that were significantly enriched or depleted in both the extended-KNN algorithm and the regularized linear model. Enrichment/depletion of a genomic feature was defined as a feature with hypergeometric test p-value P<0.01 in all cross-validations. (PDF) [file pgen.1003396.s020.pdf]

Table S16. Enriched and depleted genomic features in multi-SNP models

Enriched genomic features	Description / source
CDS	SNP Location in gene
3UTR	SNP Location in gene
GC-50-High	GC% > 0.7 in the flanking 50bp of the SNP
GC-100-High	GC% > 0.7 in the flanking 100bp of the SNP
HistoneAcetyl-H2AK5ac	Different Histone markers, taken from http://dir.nhlbi.nih.gov/papers/lmi/epigenomes/hgtcell.aspx
HistoneAcetyl-H2BK5ac	
HistoneAcetyl-H3K27ac	
HistoneAcetyl-H3K4ac	
HistoneAcetyl-H4K91ac	
Active_Promoter	Encode track from the paper by Ernst and Kellis: doi:10.1038/nbt.1662
Weak_Promoter	
Strong_Enhancer	
Txn_Transition	
MIRNA_PITA	SNP located in microRNA target site by PITA prediction (http://genie.weizmann.ac.il/pubs/mir07/mir07_prediction.html)
CPG	UCSC CpG Island track
TFBS_CENT	transcription factor binding sites from http://centipede.uchicago.edu/
MethylSeq_Gm12878	Methylation status, taken from ENCODE, Hudson Alpha GM12878/91/92
TSS_UP_0_1000	TSS/TES distance bin, gene coordinates taken from UCSC
TES_UP_0_1000	
TSS_DOWN_0_1000	
TSS_UP_1000_5000	
TES_UP_1000_5000	
TSS_DOWN_1000_5000	
TES_DOWN_1000_5000	
TSS_UP_5000_15000	
TES_UP_5000_15000	
TSS_DOWN_5000_15000	
TES_DOWN_5000_15000	
**Depleted genomic features**	**Description / source**
GC-50-MedLow	0.3 < GC% < 0.5 in the flanking 50bp of the SNP
GC-100-MedLow	0.3 < GC% < 0.5 in the flanking 100bp of the SNP
Heterochrom	Encode track from the paper by Ernst and Kellis: doi:10.1038/nbt.1662

shown are SNP genomic features that were significantly enriched or depleted in both the extended-KNN algorithm and the regularized linear model. Enrichment/depletion of a genomic feature was defined as a feature with hypergeometric test p-value P<0.01 in all cross-validations
