# Supplementary material for: Robust Prediction of Expression Differences among Human Individuals Using Only Genotype Information
Source: PLoS Genet. 2013 Mar 28;9(3):e1003396. doi: 10.1371/journal.pgen.1003396 (PMC3610805; doi:10.1371/journal.pgen.1003396)
Supplement: Table S17 — GO categories enrichment. Shown are the GO categories that are enriched in the set of 850 predictable genes. P-values were computed using Hypergeometric test and corrected using FDR with 5% threshold. Hypergeometric test and correction were performed by an in-house Perl script, and GO categories were downloaded from the gene ontology database (http://www.geneontology.org/). (PDF) [file pgen.1003396.s021.pdf]

GO category	P-value	#genes
cytoplasmic part	1.22E-07	143
mitochondrion	4.04E-06	51
MHC class II protein complex	5.60E-06	6
ribosome	6.98E-06	20
MHC protein complex	1.32E-05	8
MHC class II receptor activity	1.50E-05	5
structural constituent of ribosome	2.87E-05	18
antigen processing and presentation of peptide or polysaccharide antigen via MHC class II	3.20E-05	6
cytoplasm	3.89E-05	178
intracellular part	6.93E-05	291
intracellular organelle part	0.00013581	108
organelle part	0.00013581	108
antigen processing and presentation	0.00014958	9
ribonucleoprotein complex	0.00015573	29
cellular polysaccharide catabolic process	0.00017971	5
polysaccharide catabolic process	0.00017971	5
organelle membrane	0.00038388	39
RNA polymerase complex	0.00073221	6
